# Hepatobiliary Tumor Organoids Reveal HLA Class I Neoantigen Landscape and Antitumoral Activity of Neoantigen Peptide Enhanced with Immune Checkpoint Inhibitors

**DOI:** 10.1002/advs.202105810

**Published:** 2022-06-05

**Authors:** Wenwen Wang, Tinggan Yuan, Lili Ma, Yanjing Zhu, Jinxia Bao, Xiaofang Zhao, Yan Zhao, Yali Zong, Yani Zhang, Shuai Yang, Xinyao Qiu, Siyun Shen, Rui Wu, Tong Wu, Hongyang Wang, Dong Gao, Peng Wang, Lei Chen

**Affiliations:** ^1^ Fudan University Shanghai Cancer Center Department of Oncology Shanghai Medical College Fudan University Shanghai 200032 China; ^2^ School of Life Science and Technology ShanghaiTech University Shanghai 201210 China; ^3^ CAS Key Laboratory of Computational Biology Shanghai Institute of Nutrition and Health Shanghai Institutes for Biological Sciences Chinese Academy of Sciences Shanghai 200031 China; ^4^ University of Chinese Academy of Sciences Beijing 100049 China; ^5^ The International Cooperation Laboratory on Signal Transduction Eastern Hepatobiliary Surgery Hospital Second Military Medical University Shanghai 200438 China; ^6^ National Center for Liver Cancer Shanghai 200441 China; ^7^ School of Medicine Nanjing University Nanjing 210093 China; ^8^ Institute of Metabolism and Integrative Biology Fudan University Shanghai 200433 China; ^9^ Department of Biliary Surgery I Eastern Hepatobiliary Surgery Hospital Second Military Medical University Shanghai 200438 China; ^10^ State Key Laboratory of Cell Biology Shanghai Key Laboratory of Molecular Andrology Shanghai Institute of Biochemistry and Cell Biology CAS Center for Excellence in Molecular Cell Science Chinese Academy of Sciences Shanghai 200031 China; ^11^ Institute for Stem Cell and Regeneration Chinese Academy of Sciences Beijing 100101 China; ^12^ Key Laboratory of Signaling Regulation and Targeting Therapy of Liver Cancer (SMMU) Ministry of Education Shanghai 200438 China; ^13^ Shanghai Key Laboratory of Hepatobiliary Tumor Biology (EHBH) Shanghai 200438 China

**Keywords:** immune checkpoint inhibitor, multiomics analysis, neoantigen, patient derived hepatobiliary tumor organoid, TP53

## Abstract

Neoantigen‐directed therapy lacks preclinical models recapitulating neoantigen characteristics of original tumors. It is urgent to develop a platform to assess T cell response for neoantigen screening. Here, immunogenic potential of neoantigen‐peptides of tumor tissues and matched organoids (*n* = 27 pairs) are analyzed by Score tools with whole genome sequencing (WGS)‐based human leukocyte antigen (HLA)‐class‐I algorithms. The comparisons between 9203 predicted neoantigen‐peptides from 2449 mutations of tumor tissues and 9991 ones from 2637 mutations of matched organoids demonstrate that organoids preserved majority of genetic features, HLA alleles, and similar neoantigen landscape of original tumors. Higher neoantigen load is observed in tumors with early stage. Multiomics analysis combining WGS, RNA‐seq, single‐cell RNA‐seq, mass spectrometry filters out 93 candidate neoantigen‐peptides with strong immunogenic potential for functional validation in five organoids. Immunogenic peptides are defined by inducing increased CD107aCD137IFN‐*γ* expressions and IFN‐*γ* secretion of CD8 cells in flow cytometry and enzyme‐linked immunosorbent assay assays. Nine immunogenic peptides shared by at least two individuals are validated, including peptide from TP53^R90S^. Organoid killing assay confirms the antitumor activity of validated immunogenic peptide‐reactive CD8 cells, which is further enhanced in the presence of immune checkpoint inhibitors. The study characterizes HLA‐class‐I neoantigen landscape in hepatobiliary tumor, providing practical strategy with tumor organoid model for neoantigen‐peptide identification in personalized immunotherapy.

## Graphical abstract

A patient‐derived hepatobiliary tumor organoid system, preserving the neoantigens of corresponding tumor tissues, allows the functional selection of immunogenic neoantigen‐peptides from candidates predicted with multiomics sequencing analysis. A synergistic antitumor immunity effect of neoantigen‐peptide‐reactive T cells and ICIs proposes novel therapeutic strategies for hepatobiliary cancer patients.

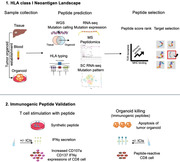

## 1 Introduction

Hepatic carcinoma is ranked as the second most lethal malignancy with only 18% of patients surviving past 5 years and over one million patients are speculated to die from liver cancer in 2030.^[^ ^1^ ^]^ Besides the scant favorable impact on the prognosis of current cancer treatments; despite the appeal of immunotherapy, there are no satisfactory survival benefits in recent clinical trials with anticytotoxic T‐lymphocyte‐associated protein 4 (CTLA4) or antiprogrammed cell death 1 (PD1) immune checkpoint inhibitor (ICI), showing the response rate of 15–20% in liver cancer.^[^ ^2^ ^]^ For current situations, it is paramount to exploit a novel promising therapy.

Neoantigens are mutant peptides predominantly from tumor‐specific nonsynonymous mutations, that can be targeted by stimulated endogenous T cells to achieve antitumor immune attack.^[^ ^3^ ^]^ Sequencing‐based neoantigen algorithms could predict thousands of candidates with high binding affinity to patients’ own human leukocyte antigen‐I (HLA‐I) molecules for each patient.^[^ ^4^ ^]^ However, the limitations of prediction‐based screening approaches are indicated in the available functional tests of solid cancers. Only a small proportion (0.5–2%) of the filtered missense single nucleotide variants (mSNVs) are validated to generate immunogenic peptides and overwhelming majority are unique to individuals.^[^ ^5^ ^]^

The development of neoantigen‐directed immunotherapies for cancers has been hindered by a shortage of in vitro preclinical models to accurately predict the efficacy of candidate peptides. The patient derived organoids (PDOs) that recapitulate primary tumors might surpass these limitations.^[^ ^6^ ^]^ On the other hand, the quantity of patients’ T cells cannot meet the huge amount of candidate neoantigen peptide validations. The potential solution is to gain T cells from different healthy individuals and test their responses to each candidate peptide, to finally identify immunogenic neoantigen peptides shared by individuals.^[^ ^7^ ^]^

Here, we provided the proof of concept that organoids maintained the comparable genetic features and neoantigen landscape of parental tumors. We integrated multiomics analysis entailing the whole genome sequencing (WGS), RNA‐sequencing (RNA‐seq), single cell (SC) RNA‐seq, and mass spectrometry (MS) to optimize neoantigen peptide prediction. Of note, by coculturing candidate peptides with HLA‐class‐I matched peripheral blood mononuclear cells (PBMCs), we obtained neoantigen peptide‐reactive T cells that kill tumor organoids and achieved the enhancement of ICIs on the T cell‐mediated tumor attack at the level of individuals. Collectively, we provided a practical strategy with organoids as an in vitro platform to assess the immunogenicity of candidate peptides, which can be applied for efficacious screening and rapid validation of neoantigen peptides for precision immunotherapy in future.

## 2 Results

### 2.1 Hepatobiliary Tumor Organoids Retain Parental Neoantigen Related Genetic Features

We have previously established long term in vitro culture of hepatobiliary cancer derived organoids.^[^ ^8^ ^]^ In our current study, using blood cells as control, we performed WGS analysis of 27 pairs of tissues and organoids, with the coverage depths of 240G for tissues and organoids, 120G for blood. First, the top 20 high frequency mutant genes of 27 paired tissues and organoids including tumor protein P53 (TP53, 70%), titin (TTN, 26%), mucin 4 (MUC4, 22%), mucin 16 (MUC16, 20%) obtained by WGS analysis were shown (Figure S1A, Supporting Information). Since personalized neoantigens predominantly derived from nonsynonymous mutations,^[^ ^3^ ^]^ then we compared the total and nonsynonymous mutations of tissues and matched organoids and found a high proportion of shared mutations and a close correlation (Figure S1B,C, Supporting Information).

**Figure 1:**
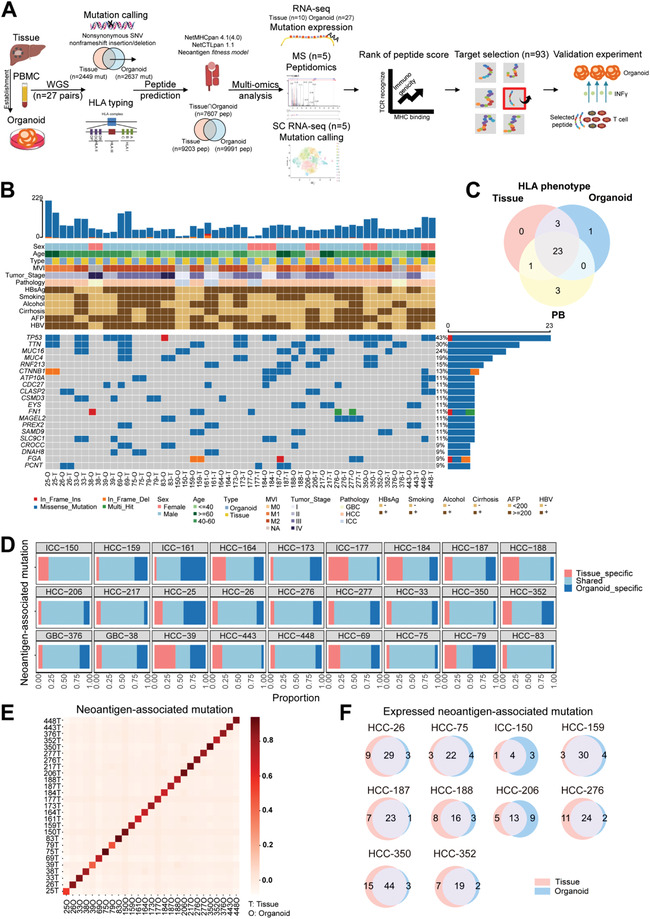
Organoids recapitulate neoantigen‐associated mutations of matched tissues. A) Schematic outlining the candidate neoantigen peptide prediction, selection, and validation. B) Data of top 20 high‐frequency neoantigen‐associated mutations predicted with WGS analysis and clinical information for all 27 patients with hepatobiliary cancer. T: tissue, O: organoid. C) Venn diagram comparing the HLA‐A, ‐B, and ‐C phenotypes between PBMCs, tissues, and organoids of individuals (*n* = 27). D) Proportions of neoantigen‐associated mutations predicted with WGS analysis detected only in tissues (red), only in organoids (dark blue), or shared (light blue) within each patient. E) Corregram indicating neoantigen‐associated mutations of each organoid correlated to its corresponding tissue, but rarely with others. Color key from dark brown to light brown indicates correlation from high to low. F) Venn diagrams showing the major concordance of all expressed neoantigen‐associated mutations confirmed with RNA‐seq between paired tissues and organoids. The area represents the mutation numbers in each segment.

Score tools previously published were used here to quantitatively assess the immunogenic potential of peptides, the detailed algorithms were described in the method part.^[^ ^9^ ^]^ As the developer mentioned that Score tools were only for the most common 9–11mer (amino acid length) peptides, in this study, we focused on 9–11mer neoantigen peptides binding to HLA‐A, ‐B, and ‐C derived from nonsynonymous single nucleotide variant (SNV) and nonframeshift insertion/deletion (InDel). Neoantigen peptides from overlapped mutations of tissue and matched organoid were predicted in WGS‐based analysis, the mutations with potential capability to generate neoantigen peptides are termed as “neoantigen‐associated mutations.” According to the expressions of corresponding mutated transcripts evaluated by RNA‐Seq analysis, peptides from unexpressed mutations were excluded. Meanwhile candidate neoantigen peptides were screened with MS and predicted neoantigen‐associated mutations were screened at single cell RNA level with SC analysis. Detailed workflow of bioinformatics analysis and validation experiment was shown in **Figure** **1**A.

Whether organoids preserve corresponding tissues’ neoantigen‐associated mutations is a key factor in evaluating the capacity of tumor organoids as a platform to identify patients’ neoantigen peptides. Here, we analyzed and compared the neoantigen‐associated mutations of paired tissues and organoids. The clinical characteristics of 27 patients (22 hepatocellular carcinoma (HCC), 3 intrahepatic cholangiocarcinoma (ICC), and 2 gallbladder cancer (GBC)) and top 20 high‐frequency neoantigen‐associated mutant genes in WGS analysis (including TP53 (43%), TTN (30%), MUC16 (24%), MUC4 (19%)) were shown (Figure 1B; and Table S1, Supporting Information).

To evaluate whether organoids retain the immunogenicity of tumor tissues, considering the essential of HLA restriction involved in the T cell stimulation and antitumor immune attack, we analyzed the HLA phenotypes of individuals. Using the HLA phenotypes of patients’ PBMCs as control, we observed HLA alterations of tissues continuously represented in the organoids and found that the HLA‐A, ‐B, and ‐C alleles of organoids were mostly consistent with those of corresponding tissues, except HCC 75 (Figure 1C). Currently, the reason for this difference is unknown. This phenomenon is possibly caused by some computational or methodological artifact. HLA target sequencing could be complementarily used to improve the accuracy of HLA typing in future. In our study, the overlapped HLA‐class‐I alleles generated from three tools in both tissues and matched organoids were used for neoantigen analysis. The detailed HLA‐A, ‐B, and ‐C information was shown in Table S1 (Supporting Information).

Subsequently the mutations of organoids and corresponding tissues were further compared. 66.73% of neoantigen‐associated mutations (range: 28.57–88.89%) were shared by organoids and matching tissues on average (Figure 1D). Since the sections for sequencing and organoid establishment were different, the low frequencies of shared neoantigen‐associated mutations in certain cases could be attributed to intratumor heterogeneity and sample acquisition. Neoantigen‐associated mutation correlation analysis further indicated that each organoid correlated to its corresponding original tissue, but rarely with other tissues (Figure 1E).

Considering mutational expression level is a key determinant of immunogenicity for neoantigens generated from nonsynonymous mutations,^[^ ^10^ ^]^ we compared the mutated transcripts of ten organoids and corresponding tumor tissues by RNA‐seq analysis and found a large overlap of expressed neoantigen‐associated mutations shared by paired organoids and tissues, verifying the neoantigen‐associated mutations of original tissues maintained in organoids at transcription level (Figure 1F).

Overall, these results demonstrated that tumor organoids recapitulated neoantigen related gene variations of the primary tissues and maintained patient‐specific heterogeneous neoantigen profiles.

### 2.2 Higher Neoantigen Load Correlated with Early Tumor Stage

**Figure 2:**
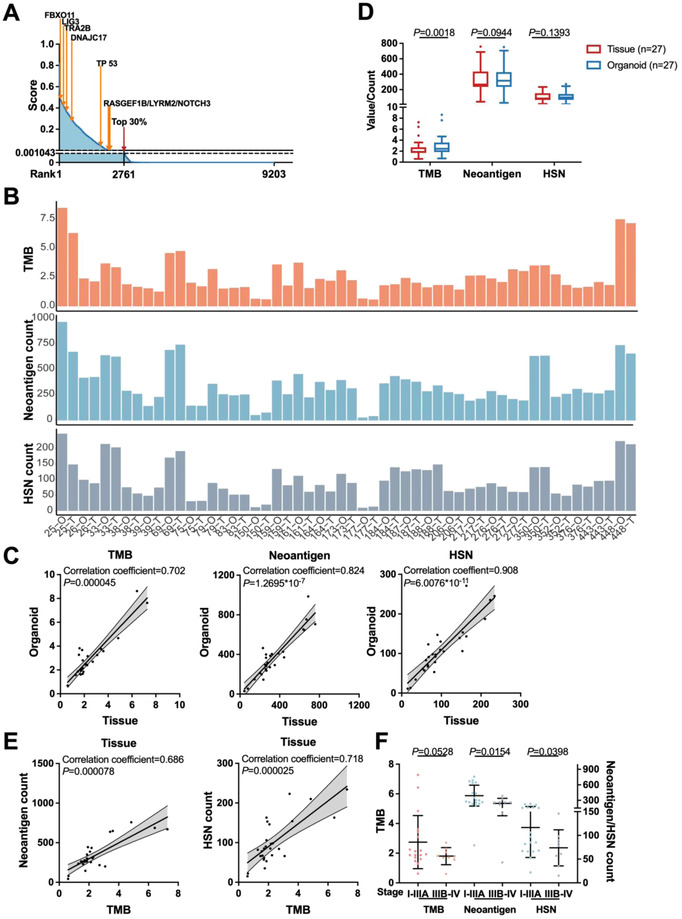
Correlations between TMB/neoantigen/HSN and tumor stage in hepatobiliary cancer. A) Immunogenic potential‐related score rank for all 9203 predicted neoantigen peptides in tumor tissues. Peptides were ordered from highest to lowest score, with the cutoff (0.001 043) for HSNs (high score neoantigen peptides) highlighted with red arrow, and the scores of shared immunogenic peptides highlighted with orange arrows. B) Values of TMB, counts of neoantigens, and HSNs for each patient. T: tissue, O: organoid. C) Positive correlations of TMB, the load of neoantigens/HSNs between organoids and tissues (*n* = 27 pairs). *P*‐values were estimated by Spearman's Rho. D) Comparisons of TMB, neoantigen, and HSN load between tissues and organoids (*n* = 27 pairs). *P*‐values were calculated by two‐tailed paired *t*‐test. E) Positive correlations between the load of neoantigens/HSNs and TMB in tissue (*n* = 27). *P*‐values were estimated by Spearman's Rho. F) Neoantigen and HSN load, rather than TMB correlated with patients’ tumor stage (18 stage I‐IIIA, 9 stage IIIB‐IV). Data are presented as mean ± SD, *P*‐values were calculated by two‐tailed unpaired *t*‐test.

The immunogenic potential‐related scores of total 9203 WGS analysis‐based predicted neoantigen peptides of tissues were ranked (median 2.38 × 10^−7^, range 0–0.82), and top 30% peptides were over 0.001 043 (**Figure** **2**A). To analyze the features of predicted neoantigen peptides with relatively high scores, we temporarily termed peptides with scores over 0.001 043 as high score neoantigen peptides (HSNs) in subsequent analysis.

Tumor mutational burden (TMB) is a recognized effect predictive marker of ICIs.^[^ ^11^ ^]^ To assess the clinical predictive value of neoantigen load, we compared TMB and neoantigen load, and found the consistent trend of TMB, counts of predicted neoantigen/HSN peptides of both tissues and organoids in individuals (Figure 2B). Significantly positive correlations of both mutation and neoantigen burden between tissues and organoids established organoid as the representative of matched tumor tissue in neoantigen study (Figure 2C). In comparison with the tissues, the organoids harbored higher TMB (Figure 2D). The tumor purity of organoids was relatively higher than that of tissues basing on WGS analysis (Figure S2A, Supporting Information). The malignant percentages of organoid cells were almost 100% in single‐cell analysis (Figure S2B, Supporting Information). Since tumor cellularity was reported to be positively related to TMB.^[^ ^12^ ^]^ Hence, we think reasons for the low TMB of the tissues could arise from either technical (such as sequencing coverage depth) or biological causes (such as sample acquisition, tumor heterogeneity, and tumor purity). The neoantigen load of organoids was higher than that of tissues, but no significant difference was found (Figure 2D). Then we calculated and detected significantly positive correlations between TMB and neoantigen load in tissues (Figure 2E), implying the predictive value of neoantigen load in ICI therapeutic effect of hepatobiliary cancer.

To explore whether mutation and neoantigen burden were associated with patients’ clinical characteristics, the patients of early (I‐IIIA) and late (IIIB‐IV) tumor node metastasis (TNM) stages were compared. Higher TMB and neoantigen burden were found in patients of early tumor stage, but significant difference was observed only when total neoantigen or HSN load was compared (Figure 2F). Additional analysis was performed to analyze the relationships between TMB/neoantigen load and sex, age, microvascular invasion (MVI), hepatitis B surface antigen (HBSAg), smoking, alcohol, cirrhosis, serum alpha‐fetoprotein (AFP) status (Figure S3, Supporting Information). Neoantigen/HSN load was significantly higher in the patients with high AFP level. HSN load and TMB were significantly higher in older patients and patients with MVI M0. These results suggested the clinical value of lower neoantigen load to predict tumor malignancy for patients with hepatobiliary cancer. A large dataset of patients will be future studied to confirm this conclusion.

### 2.3 Hepatobiliary Tumor Organoids Recapitulate HLA Class I Neoantigen Features of Primary Tumors

**Figure 3:**
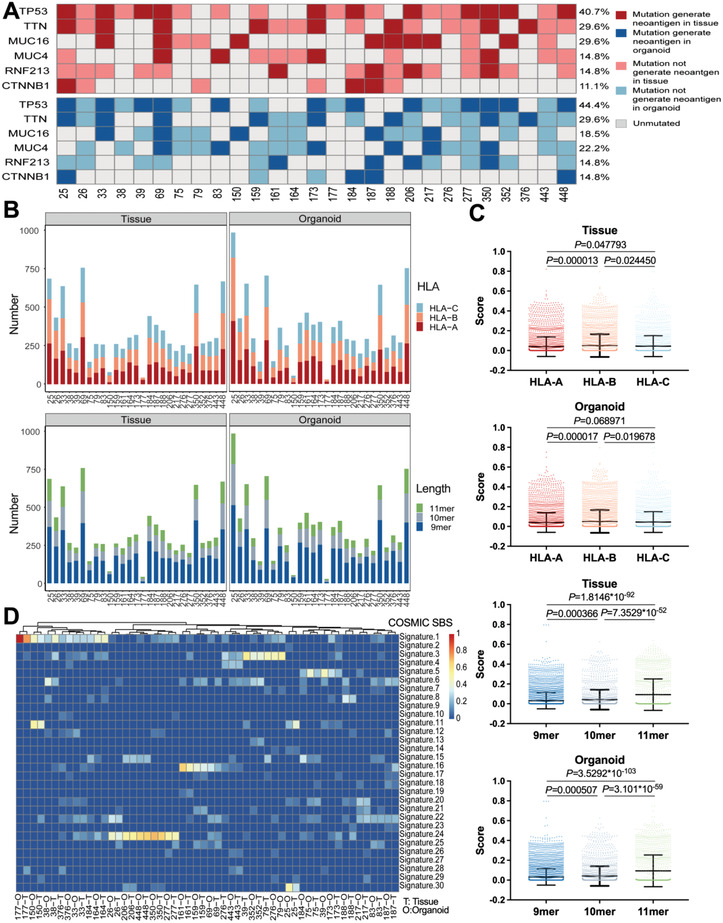
Organoids recapitulate predicted neoantigen peptide features of matched tissues. A) Top high frequency neoantigen‐associated mutations shared by patients in tissues and organoids. B) Counts of predicted neoantigen peptides with each HLA allele for individual tissues and organoids. Counts of predicted neoantigen peptides with each length for individual tissues and organoids. C) The immunogenic potential‐related scores of predicted peptides with each length and HLA allele were compared (tissue: 3264 HLA‐A binding peptides, 3118 HLA‐B binding peptides, 2821 HLA‐C binding peptides; organoid: 3566 HLA‐A binding peptides, 3402 HLA‐B binding peptides, 3023 HLA‐C binding peptides; tissue: 5308 9mer length peptides, 2270 10mer length peptides, 1625 11mer length peptides; organoid: 5718 9mer length peptides, 2474 10mer length peptides, 1799 11mer length peptides). Data are presented as mean ± SD, *P*‐values were calculated with ANOVA followed by post‐hoc test (LSD) to compared multiple groups. The immunogenic potential‐related scores showed significantly difference among three subgroups (for tissues: *P* = 0.000 072 among HLA‐A HLA‐B and HLA‐C binding peptide subgroups, *P* = 7.1731 × 10^−92^ among 9mer, 10mer, and 11mer peptide subgroups; for organoids: *P* = 0.000 091 among HLA‐A, HLA‐B, and HLA‐C binding peptide subgroups, *P* = 5.0223 × 10^−103^ among 9mer, 10mer, and 11mer peptide subgroups). D) Neoantigen‐associated mutational COSMIC SBS Signatures of paired tissues and organoids for 27 hepatobiliary cancer patients. The relative contribution of each signature was calculated with R package deconstructSigs. Color key from red to blue indicates relative contribution from high to low.

First, we focused on the neoantigen‐associated mutations shared by individuals in our prediction. Two mutant genes with frequency over 25% (TP53 (44.4%) and TTN (29.6%)) were observed in organoids; in agreement with the high frequency neoantigen‐associated mutant genes observed in the original tumor tissues (**Figure** **3**A). Notably, TP53 mutation also ranked as the top one high frequency mutation in WGS analysis, suggesting the vast share of TP53 mutation‐associated neoantigen among hepatobiliary cancer patients. Building a library of validated immunogenic neoantigen peptides shared by different individuals ready for clinical use is a timely and efficient therapeutic strategy for cancer patients, especially those advanced tumors with rapid progress. With the aim to complement the neoantigen peptide library, the immunogenicity of peptides arising from TP53 mutation were concerned in later immunogenicity evaluations.

Second, the pattern comparison confirmed that organoids preserved the candidate neoantigen features of matching tissues. When evaluating total peptides derived from nonsynonymous SNV and nonframeshift InDel, we found that candidate peptides were significantly enriched in the subset with the length of 9mer, whereas no obvious difference in the distribution of HLA‐A, ‐B, and ‐C alleles (Figure 3B; and Figure S4A,B, Supporting Information); moreover, the immunogenic potential‐related scores of peptides with 11mer and binding to HLA‐B were significantly highest in both organoids and tissues (Figure 3C). In SNV class analysis, neoantigen‐associated mutations were characterized predominantly by C>T and G>A mutations (Figure S4C,D, Supporting Information). Then we analyzed Catalogue of Somatic Mutations in Cancer (COSMIC) single base substitutions (SBS) mutational signatures and found that the signatures of organoids and paired tissues were highly consistent and both enriched with COSMIC SBS Signature 1, 3, 5, 16, and particularly COSMIC SBS Signature 24 (http://cancer.sanger.ac.uk/cosmic/signatures, Figure 3D). Taken together, organoids maintained the HLA‐class‐I neoantigen features of the primary tissues.

### 2.4 Identification of Immunogenic Neoantigen Peptides

**Figure 4:**
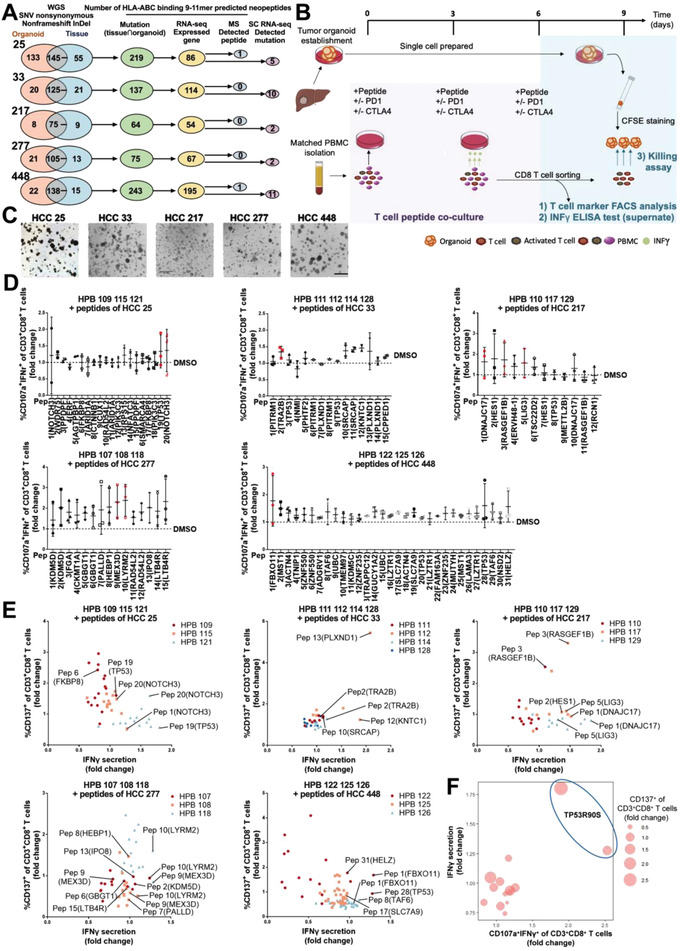
Identification of immunogenic peptides by peptide‐T cell coculture. A) In five cases, the numbers of WGS‐detected SNV nonsynonymous, nonframeshift insertion/deletion (InDel) in organoids and paired tissues; the numbers of predicted HLA‐A, ‐B, and ‐C binding 9–11mer peptides derived from overlapped mutations of organoids and tissues, from RNA‐seq detected expressed mutations, from MS‐detected peptide database; the numbers of expressed mutations detected with single cell RNA‐seq were shown. B) Validation experimental workflow. PBMCs were stimulated with peptides for three cycles. After 9 days of coculture, T cell activation markers and IFN‐*γ* production were assessed using flow cytometry and ELISA, CD8^+^ T cell effector function was evaluated in killing assay after another 3‐day coculture with organoids. C) Bright field images of five tumor organoids, scale bar: 560 µm. D) dimethyl sulfoxide (DMSO) treatment was used as control and fold changes of peptide induced CD107a^+^IFN‐*γ* ^+^ coexpression of CD3^+^CD8^+^ T cells after three cycles of stimulation were quantified. Dots indicate independent experiments with healthy peripheral blood (HPB) from different donors. Data are presented as mean ± SD. Immunogenic peptides shared by donors were marked in red. E) DMSO treatment was used as control and fold changes of peptide induced IFN‐*γ* secretion and CD137^+^ expression of CD3^+^CD8^+^ T cells after three cycles of stimulation were quantified. Dots indicate independent experiments with HPB from different donors. Immunogenic peptides judged by CD107a^+^IFN‐*γ* ^+^ coexpression levels were annotated. F) Scatterplot of the immunogenicity of TP53 mutation‐derived peptides tested for fold changes of CD107a^+^IFN‐*γ* ^+^ coexpression of CD3^+^CD8^+^ T cells (*x* axis) versus fold changes of IFN‐*γ* secretion (*y* axis). Dot size represents fold changes of CD137^+^ expression of CD3^+^CD8^+^ T cells.

To evaluate the immunogenicity of predicted peptides with relatively high immunogenic potential‐related scores, five established HCC organoids with stable proliferative ability were respectively analyzed. We established a multiomics approach to filter HLA‐A, ‐B, and ‐C binding candidate peptides derived from nonsynonymous SNV and nonframeshift InDel. On average, 148 peptides with 9–11mer length derived from 118 overlapped mutations of individual organoids and matching tissues were predicted by WGS analysis, then the peptides with no detectably expression of the corresponding mutant transcripts in RNA‐seq were excluded. In order to explore the contributions of MS and single cell RNA‐seq to neoantigen peptide prediction, we utilized these two tools to screen the candidate peptides. Only two peptides were detected by MS and 30 neoantigen‐associated mutations were detected by single cell RNA‐seq (**Figure** **4**A; and Figure S5, Supporting Information). The extremely low number of peptides detected by MS may be due to the limited sensitivity of this technique. Sensitive MS‐based immunopeptidomics with advanced bioinformatics tools are expected to uncover the full landscape of neoantigen peptides. The limitation in coverage depth of 10× single cell RNA‐seq could partly explain a small number of neoantigen‐associated mutations detected with SC analysis.

To validate the immunogenic peptides, we performed T cell stimulation with peptides and organoid killing assay of peptide‐reactive T cells. We ranked predicted peptides by the immunogenicity‐related scores and selected peptides with relatively high scores. We preferentially selected peptides detected with MS and TP53 mutation‐derived peptides. For each patient, at least 10 peptides binding to HLA‐class‐I alleles were selected for validation. A total of 93 candidate peptides was selected. HLA‐A, ‐B, and ‐C at least half matched healthy PBMCs (except HPB 128, due to lack of HLA‐A*32:01 blood) were used in the peptide function assays (Tables S2 and S3, Supporting Information). Based on IFN‐*γ* intracellular expression and secretion into coculture supernatants, the degranulation marker CD107a, and the activation marker CD137 upon stimulation; the immunogenicity of peptides was comprehensively evaluated. Because the CD107a^+^IFN‐*γ* ^+^CD3^+^CD8^+^ cells obviously existed in control (T cells treated with dimethyl sulfoxide (DMSO), Figure S6, Supporting Information), we preferred the fold change instead of exact frequency to judge the immunogenicity of peptides. The responses of T cells from different donors under the same peptides varied, to ensure the accuracy of immunogenicity judgement, immunogenic peptides after three cycles of peptide‐T cell coculture were strictly defined by different criteria as follows: 1) at least 1.5‐fold higher frequency of CD107a^+^IFN‐*γ* ^+^ cells in CD3^+^CD8^+^ cells compared with negative controls; 2) if half peptides over 1.5‐fold change, the cutoff could be reset to 2‐ or 3‐fold change; 3) if no peptide over 1.5‐fold change, the top peptide was selected. The reactive CD8 T cells against the pools of validated immunogenic neoantigen peptides were further expanded for organoid killing assays (Figure 4B). Bright field images of these five tumor organoids were shown in Figure 4C; and Figure S7 (Supporting Information). Both technical and biological replicates (HLA matched PBMCs from different healthy donors) were performed in the analysis of T cell reactivity against peptides. We presented the immunogenicity per peptide as the average of all replicates (Figure 4D). The detailed fold changes of each peptide were indicated and the immunogenic peptides judged with our criteria were marked with red box in Figure S6 (Supporting Information). T cells stimulated with immunogenic peptides showed relatively high IFN‐*γ* secretion and CD137 expression, further confirmed the immunogenicity of these peptides (immunogenic peptides annotated in Figure 4E).

T cell responses under 93 peptide treatments were, respectively, assessed and the immunogenicity of 25 peptides were validated (Table S4, Supporting Information). Mainly discrepancy, but still consistency was observed in peptide tests among individuals. T cell reactivities were consistently observed when T cells from different healthy donors were stimulated with peptide 19 20 of HCC 25, peptide 2 of HCC 33, peptide 1 3 5 of HCC 217, peptide 9 10 of HCC 217, peptide 1 of HCC 448 (red in Figure 4D; and Figure S6, Supporting Information). We speculated that these peptides (named as shared immunogenic peptides) are most likely to be neoantigen peptides for clinical therapy of cancer patients.

Then, we focused on TP53 mutation‐derived peptides among 93 candidates. Interestingly, HCC 33 (TP53 R81L R54L), HCC 217 (TP53 S81C), and HCC 448 (TP53 R210S) did not have T cell responses to TP53 mutation‐derived peptides. In contrast, T cells were reactive to the TP53 mutation‐derived peptides in HCC 25 (TP53 R90S) and HCC 448 (TP53 R90S) with HLA‐C binding (Figure 4F). We surmised peptides derived from TP53 R90S were likely immunogenic outside of other TP53 genetic changes. We next questioned whether peptides detected by single cell analysis or MS behaved stronger immunogenicity. Seven peptides derived from mutations detectable in single cell RNA‐seq (43.8% of total tested) induced T cell reactivity, including HCC 25 (FKBP prolyl lsomerasem8, FKBP8), HCC 33 (transformer 2 beta homolog, TRA2B), HCC 217 (hes family BHLH transcription factor 1, HES1), HCC 277 (heme binding protein 1, HEBP1; LYR motif containing 2, LYRM2), and HCC 448 (TP53; helicase with zinc finger, HELZ) (Tables S3 and S4, Supporting Information). This highly accurate prediction reflected the future value of single cell analysis especially the whole transcriptome based single cell sequencing in neoantigen prediction. Unfortunately, neither of the two peptides detected by MS possessed immunogenicity (Table S3, Supporting Information), implying the weakness of neoantigen prediction and the significance of immunogenicity validation experiment.

### 2.5 Impact of ICI Treatment on T Cell Stimulation

**Figure 5:**
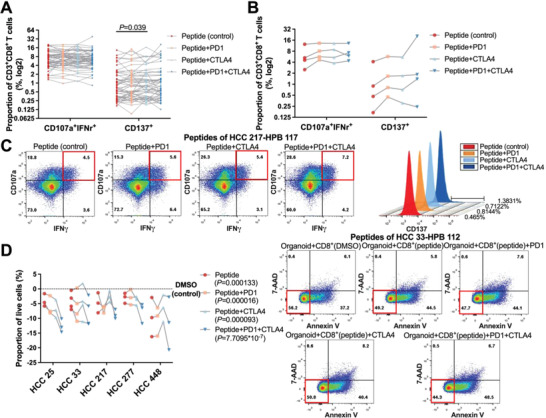
T cell reactivity with ICI treatment and tumor organoids killed by immunogenic peptide‐reactive T cells. A) CD107a IFN‐*γ* CD137 expressions of CD3^+^CD8^+^ T cells from different individuals stimulated by HLA‐A, ‐B, or ‐C peptide pool and treated with ICIs or not were determined by flow cytometry. Lines present different cases (*n* = 54). *P*‐values were calculated by two‐tailed paired *t*‐test. B) In four cases, ICI treatment increased the CD107a IFN‐*γ* CD137 expressions of CD3^+^CD8^+^ T cells stimulated with peptide pool. Lines present different cases (*n* = 4). C) Representative flow cytometry plots gated on CD3^+^CD8^+^ T cells treated with ICIs or not under peptide stimulation. D) The organoid killing efficiencies of peptide‐reactive T cell cocultured with or without ICIs were compared by the proportion of live cells (Annexin V^–^7‐AAD^−^) with flow cytometry. The viability of organoids cocultured with DMSO was used as control and set to zero. Lines present different individuals (*n* = 16). *P*‐values were calculated by two‐tailed paired *t*‐test. Representative flow cytometry plots gated on CD45^−^CFSE(FITC)^+^ organoids.

To test whether ICIs play a role on T cell stimulation, we compared T cell marker expressions upon three‐cycle stimulation of peptides that were either treated with ICIs or untreated as control (Figure 4B). PBMCs from different healthy donors were, respectively, stimulated with corresponding HLA‐A, ‐B, or ‐C binding peptides of different HCC organoids. CTLA4 exposure led to a significant CD137 upregulation in CD8^+^ T cells, no other significant difference was observed regardless of ICIs application (**Figure** **5**A). Then we checked each case and found that in four of 54 tested (HPB 115 treated with HLA‐C*03:04 peptides of HCC 25, HPB 117 treated with HLA‐C*03:02 peptides of HCC 217, HPB 118 treated with HLA‐A*11:01 peptide 6–10 of HCC 277, HPB 126 treated with HLA‐A*11:01 peptides of HCC 448), ICIs treatment had an effect of improving IFN‐*γ* CD107a and CD137 in CD8^+^ T cells, but the effect of IFN‐*γ* CD107a was limited (Figure 5B,C). The limited upregulation indicated that the activation of peptide‐reactive T cell response under ICIs could be induced or boosted in a minority population.

### 2.6 Tumor Organoids Killed by Neoantigen Peptide Reactive T Cells and Synergy of ICIs

Next, we wished to evaluate whether peptide‐reactive T cells were able to eradicate tumor cells. To determine this, CD8 T cells stimulated with validated immunogenic peptides or DMSO as control were sorted and cocultured with carboxyfluorescein diacetate succinimidyl ester (CFSE) labeled tumor organoids for 3 days (Figure 4B). For all samples tested, exposure to peptide‐reactive T cells significantly reduced the proportion of live organoid cells (Figure 5D). Collectively, peptide‐reactive T cells’ tumor killing effect support the feasibility of our platform to distinguish neoantigen peptides.

To assess the effect of ICIs on the tumor destruction, we applied parallel cytotoxicity assays with ICI treatments. Organoids were continuously efficiently killed by T cells after exposed to PD1+CTLA4 inhibitors, but the solely additional use of PD1 inhibitor in two samples and CTLA4 inhibitor in eight samples did not negatively affect the survival of organoids (Figure 5D). Taken together, organoids as targets can be used to measure T cell mediated antitumor effect and ICIs increased the sensitivity of tumor cells to neoantigen peptide‐reactive T cells.

### 2.7 Features of Immunogenic Neoantigen Peptides

**Figure 6:**
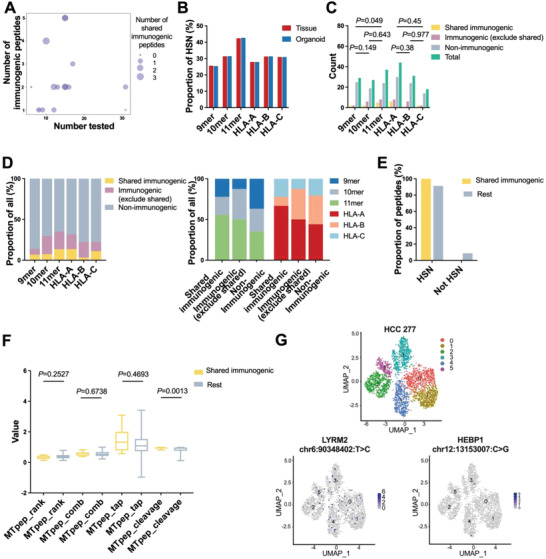
Features of validated immunogenic peptides. A) Scatterplot of peptide number tested for immunogenicity (*x* axis) versus validated immunogenic peptide number (*y* axis). Dot size represents the number of shared immunogenic peptides. B) Proportions of HSNs (high score neoantigen peptides) with each length and HLA allele in tissues and organoids. C) Counts of shared immunogenic, immunogenic (exclude shared immunogenic peptides), nonimmunogenic, and total tested peptides for each peptide length and HLA allele separately. *P*‐values were calculated with Pearson's chi‐squared test to compare the enrichment of immunogenic peptides with different length and HLA allele. D) Proportions of shared immunogenic, immunogenic (exclude shared immunogenic peptides) and nonimmunogenic peptides for each peptide length and HLA allele separately. Proportions of each peptide length and HLA allele for shared immunogenic, immunogenic and nonimmunogenic peptides separately. E) Proportions of HSNs in shared immunogenic and rest peptides. F) The scores of mutated peptide rank, cleavage, and transporter associated with antigen processing (TAP) transport combined and respective abilities of peptides stratified by immunogenicity were compared. *P*‐values were calculated with two‐tailed unpaired *t*‐test. G) UMAP plot of all single cells for HCC 277 reveals different clusters. UMAP plot of all single cells colored by different neoantigen‐associated mutations.

Characterizing the features of verified immunogenic peptides provides clue to identify immunogenic peptides. Based on the database of immunogenic peptides validated in our study, we assessed whether the characteristics differed between immunogenic and nonimmunogenic subsets. Each individual had a mean of 15 peptides tested of which on average 2.25 were immunogenic peptides, 1.25 were shared immunogenic peptides. The scattered distribution reflected the heterogeneity of immunogenicity among individuals, emphasizing the importance of peptide selection (**Figure** **6**A).

According to our pool of predicted peptides, HSNs were enriched in the subset of 11mer peptides, but no obvious difference of HLA‐A, ‐B, and ‐C distribution (Figure 6B). The most common length of predicted peptides is 9mer (Figure 3B), while the enrichment of HSNs was observed in 11mer peptides. To clarify the underlying explanation, we compared the predicted scores of peptide presentation features of 9mer and 11mer length peptides in both tissues and organoids. Compared to 9mer peptides, 11mer peptides were with significantly stronger mutated peptide binding affinity, proteasomal C' terminal cleavage and transporter associated with antigen processing (TAP) transport combined efficiency in algorithm‐based analysis (Figure S8, Supporting Information). The advantageous features of 11mer peptides explained the higher immunogenicity‐related score of 11mer peptides.

Most peptides tested were HLA‐A binding and 11mer length (Figure 6C). After comparing immunogenic and nonimmunogenic peptides with 9mer and 11mer length, we found significant enrichment of immunogenic peptides with 11mer length. Compared to peptides with 10mer length, peptides with 11mer length possessed higher frequency of immunogenic peptides, but not significantly. A trend of immunogenic peptides with HLA‐A binding was also observed (Figure 6C,D; and Table S5, Supporting Information). Then we focused on the shared immunogenic peptides, all these peptides belong to HSNs (Figure 6E), much higher than the reported proportion of immunogenic mSNVs in total,^[^ ^5^ ^]^ implying the advantage of prediction algorithm with Score tools and the feasibility of our cutoff value to define HSN. The antitumoral T cell response was associated with effective MHC binding affinity, proteasomal C’ terminal cleavage and TAP transport efficiency.^[^ ^9^ ^]^ Thus, the predicted scores of peptide presentation features were compared. Shared immunogenic peptides had higher rank and stronger cleavage and TAP transport abilities, but significance was only found while comparing cleavage ability, possibly because of the limited cases we performed (Figure 6F).

Then we focused on the neoantigen‐associated mutations detectable at single cell level in SC RNA‐seq analysis. After investigating cells carrying nonshared immunogenic peptide‐related mutation (HEBP1) and cells carrying shared immunogenic peptide‐related mutation (LYRM2) in HCC 277, we observed that cells with LYRM2 mutation exhibited relatively high frequency and broad distribution at single cell level (Figure 6G). After investigating cells with immunogenic peptide‐related mutation (HES1) and nonimmunogenic peptide‐related mutation (reticulocalbin 1, RCN1), similar situation was observed in HCC 217 that cells with immunogenic peptide‐related mutation exhibited relatively high frequency (Figure S9, Supporting Information). Mutational pattern at single cell level correlates with the potential of mutation to generate neoantigen. However, cells with mutant ribosomal protein S15 (RPS15) in HCC 25 and transmembrane protein 97 (TMEM97) in HCC 448 exhibited relatively high frequency, but peptides derived from these mutations failed to stimulate T cell response in validation experiment (Figure S9, Supporting Information). Since the evolution of cancers from normal original cells requires progressively accumulative mutations, that evade immune recognition and attack;^[^ ^13^ ^]^ the explanation for this could be that the widespread of tumor cells carrying certain mutations may be the result of immune evasion, in other words, these mutations have none or minimal potential to be neoantigens.

All in all, these features of validated immunogenic peptides in hepatobiliary cancer directed a convenience approach to select potential neoantigen peptides.

## 3 Discussion

Current sequencing, immunopeptidomics techniques and computational tools enable rapid mutation calling and neoantigen prediction from individual tumor patients.^[^ ^14^ ^]^ However, the identification of neoantigen peptides from a large pool of candidates remains a major challenge on the way to efficiently exploit each patient's unique set for later targeted immunotherapy. Here, we provide evidence of that tumor organoids can be used as a reliable preclinical in vitro model system to evaluate the immunogenicity of tumor specific peptides.

Our findings were consistent with previous studies that organoids cultured with 3D systems could recapitulate genetic complexity of original tumors even after long‐term expansion.^[^ ^6a^ ^]^ Additionally, the reproduction of neoantigen load in organoids established the proof of organoids as a platform for neoantigen peptide selection and validation, given our finding that organoids harbored around 80% neoantigen‐associated mutations of paired tissues (Figure 1D).

Tumor derived organoid was previously used as a tool to assess T cells’ antitumoral response via the contact between PBMCs and malignant cells.^[^ ^15^ ^]^ Organoid was also reported as a model to assess the effects of immunotherapies, including the anti‐HER2‐chimeric antigen receptor (CAR) T cells and neoantigen EGFRvIII‐specific CAR NK cells in colorectal cancer.^[^ ^16^ ^]^ In our study, five patient derived organoids distinctly attacked by peptide‐reactive T cells confirmed organoids as killing targets to assess the effect of neoantigen‐directed therapy. Meanwhile, the feasibility of our peptide‐T cell coculture system for immunogenic peptide identification is validated.

Across various cancers including those with low TMB, tumor specific mSNV could be recognized by tumor infiltrating lymphocytes (TILs) among over 70% patients.^[^ ^5^, ^17^ ^]^ In our organoid‐based validation experiment, at least one shared immunogenic peptide recognized by T cells was detected for each patient. These studies ensured the practicality of mSNV‐derived neoantigen peptide in T cell meditated tumor destruction. It comes another question, how to rapidly select neoantigen peptides and improve the efficiency of neoantigen‐directed therapies, several strategies were proposed here to answer this question.

To our knowledge, single cell RNA‐seq has not been reported to be complementarily utilized in the multiomics analysis for neoantigen prediction. In 25 validated immunogenic peptides, seven related mutations were detected at single cell level (Table S4, Supporting Information). Considering the limitation of sequencing coverage depth we performed here,^[^ ^18^ ^]^ the contribution of single cell analysis in neoantigen prediction is still expectable.

Clarifying the unique features of neoantigen peptides contributes to the optimization of peptide prediction.^[^ ^10^, ^19^ ^]^ Our proved immunogenic peptides were most common with HLA‐A binding and 11mer length (Table S4, Supporting Information). The subset of 11mer HLA‐A binding peptides with relatively high immunogenic potential should be emphasized especially among patients only with weak predicted immunogenic peptides. As expected, shared immunogenic peptides were not completely with the highest scores (the immunogenic potential‐related scores of shared peptides marked in Figure 2A), implying regardless of algorithm‐based prediction accuracy, the immunogenicity of peptides still requires in vitro model‐based functional validation.

A vast majority of mSNV‐derived neoantigens was proved to be unique to patients,^[^ ^5^ ^]^ thus building a library of validated neoantigen peptides shared by patients would improve the efficiency of clinical peptide selection. Immunogenic hotspot neoantigens should be prioritized, as they are theoretically likely to be clonal and stable.^[^ ^3^, ^20^ ^]^ Previous studies covering different tumor types identified the T cell recognition of TP53 and KRAS hotspot epitopes, meanwhile the T cell receptor (TCR) library restricted to different HLA molecules against hotspots was created.^[^ ^21^ ^]^ Based on the concept of library, our study also tested T cell activation upon encountering each TP53 mutation‐derived peptide, the immunogenicity of two peptides was validated. To further expand the TP53 and other mutation‐associated neoantigen peptide library, our screening model will be utilized among a wide range of patients.

ICIs emerge as the T cell inhibitory signaling blocker via directing against molecules expressed on the surface of T cells, exemplifying PD1, or CTLA4, to regress tumors.^[^ ^22^ ^]^ The close association between ICI response and mutation burden, neoantigen load led to the speculation that tumors might be easily targeted by T cells released by ICIs in neoantigen‐directed therapy.^[^ ^11^, ^23^ ^]^ The effectiveness of the combination of ICIs and neoantigen to eradicate tumor was confirmed in others’ mouse study.^[^ ^24^ ^]^ In our organoid killing assay, benefit from the administration of ICIs combined with neoantigen peptides revealed the potential of ICIs to augment tumor destruction with peptide‐reactive T cells.

Neoantigen‐associated mutational pattern identification could help to select patients who benefit from neoantigen‐directed therapy. The major contributions of COSMIC SBS Signature 1 and Signature 24 to neoantigen‐associated mutational formation were observed in our study. Signature 1 is associated with most cancers, of note Signature 24 is due to aflatoxin exposure, which contributes to the development of HCC.^[^ ^25^ ^]^ Aflatoxin exposure potentially gives rise to neoantigen‐associated mutations. We speculate that hepatobiliary cancer patients under aflatoxin exposure are more likely to obtain certain neoantigens, and these patients may benefit from the immunotherapy targeting aflatoxin exposure‐related neoantigen. This speculation will be paid close attention to in future clinical studies.

There are two limitations in this study. The first is the culture medium for organoid establishment. Because of tumor heterogeneity, some tumor tissues failed to generate organoids, the culture system should be optimized. The second limitation concerns the absence of clinical comparison to validate the neoantigen peptide predicted from our organoid‐based platform.

In conclusion, this study demonstrated a novel system combining multiomics analysis‐based neoantigen peptide prediction and organoid‐based T cell functional assay to efficiently identify neoantigen peptides as personalized immunotherapy targets. Furthermore, insights into the impacts of ICIs on peptide‐reactive T cells and efforts to create peptide library will improve the therapeutic efficacy.

## 4 Experimental Section

### Human Subjects

From July 2018 to December 2019, hepatobiliary tumor resections (≈1–3 cm^3^) and peripheral blood (≈3–5 mL) from 27 patients who underwent surgical resection were obtained. From May 2020 to October 2020, peripheral blood (≈1–3 mL) from 16 healthy donors were obtained. All participants were provided with informed consent. All experiments were approved by the Ethics Committee of Eastern Hepatobiliary Surgery Hospital (Shanghai, China; approval no.: EHBHKY2018‐1‐001)). The patients’ characteristics are shown in Figure 1B; and Table S1 (Supporting Information).

### PBMC Isolation

The PBMCs were isolated with Ficoll‐Paque separation solution (GE Healthcare) by gradient centrifugation and cryopreserved with CryoStor CS10 medium (STEMCELL Technologies) in liquid nitrogen for future use.

### Tumoral Organoid Culture

Hepatobiliary cancer organoids were established as described previously.^[^ ^8^ ^]^ In detail, fresh hepatobiliary tumor resections were obtained through surgical resection. After healthy tissue was removed, specimen (≈0.5–1.5 cm^3^) was minced and digested in digestion buffer (DMEM (Gibco) with 4 mg mL^−1^ collagenase D (Roche), 0.1 mg mL^−1^ DNase I (Sigma), 2 × 10^−6^ m Y27632 (Sigma‐Aldrich), 100 µg mL^−1^ Primocin (InvivoGen)) at 37 ℃ for 30–90 min. The suspension was then filtered through 70 µm nylon cell strainer (Falcon). The pellet was collected after washed twice in cold Advanced DMEM/F12 (Gibco) and resuspended in organoid culture medium (Advanced DMEM/F12 with 1% penicillin/streptomycin, 1% Glutamax, 10 × 10^−3^ m HEPES, 100 µg mL^−1^ primocin, 1:50 B27 supplement (without vitamin A), 1.25^ ^× 10^−3^ m N‐acetyl‐L‐cysteine, 50 ng mL^−1^ mouse recombinant EGF, 100 ng mL^−1^ recombinant human FGF10, 1 ng mL^−1^ recombinant human FGF‐basic, 25 ng mL^−1^ recombinant human HGF, 10 × 10^−6^ m forskolin, 5 × 10^−6^ m A8301, 10 × 10^−6^ m Y27632, 10 × 10^−3^ m nicotinamide, 10% vol/vol Rspo‐1 conditioned medium, 30% vol/vol Wnt3a‐conditioned medium, 5% vol/vol Noggin conditioned medium). 200 µL cell suspension mixed with 150 µL cold Matrigel Basement Membrane Matrix (Corning) was cultured in 6 well suspension culture plate at 37 ℃ for 30 min. After gelation, 2 mL medium was added to each well. Established organoids were dissociated to single cells with Tryple Express (Gibco) for 5 min at 37 ℃ and passaged in fresh medium‐matrix every week. The success rate of organoid establishment from tumor tissue is around 60% in the lab. Normally, it takes 2–3 weeks to get stable expansion of organoids for subsequent studies. Organoids later than passage 5 and earlier than passage 30 were used in this study.

### DNA/RNA Extraction and Sequencing

Organoids were harvested and dissociated into single cells with Tryple Express (Gibco) for 5 min at 37 ℃. After washed twice with PBS, cell pellet was prepared for WGS and RNA‐seq. Single cell suspension in cold PBS with cell viability over 90% was prepared for single cell RNA‐seq.

DNA/RNA extraction, library construction, and WGS, RNA‐seq were performed by Genergy Biotechnology Co. Ltd. (Shanghai, China). In detail, genomic DNA was extracted from tumor tissues and organoid cells with QIAamp Fast DNA tissue kit (QIAGEN), matched blood DNA was extracted with QIAamp DNA blood Midi Kit (QIAGEN). The libraries were sequenced by Illumina Novaseq 6000 platform following the manufacturer's instructions (Illumina Inc., San Diego, CA), with the coverage depths of 240G for tissue and organoid, 120G for blood in WGS.

Total RNA was extracted from tumor tissues and organoid cells with Trizol reagent (Invitrogen). After reverse transcription, the cDNA libraries were sequenced with the paired‐end sequencing mode of the Illumina Hiseq sequencing platform following the manufacturer's instructions (Illumina Inc., San Diego, CA), with the coverage depths of 10–12G in RNA‐seq.

Single cell RNA‐seq was performed as described previously.^[^ ^8^ ^]^ In brief, organoid cells were loaded onto the 10X Genomics Platform (Single Cell 3’ library and Gel Bead Kit v.3). Generation of gel beads in emulsion (GEMs), barcoding, GEM‐RT clean up, reverse transcription, cDNA amplification, a 3’ gene library construction were all performed. The final library pool was sequenced with the 150‐base‐pair paired‐end sequencing mode on the 10X single‐cell Illumina sequencing platform (Illumina Inc., San Diego, CA).

### Sequencing Analysis—WGS Data Analysis—Somatic Mutation Calling

Skewer (v0.2.2, https://sourceforge.net/projects/skewer) was used to remove adapter sequence fragments and low‐quality fragments from the sequencing data. FastQC (v0.11.5, http://www.bioinformatics.babraham.ac.uk/projects/fastqc) was used to perform quality control analysis on the preprocessed data. FASTQ files of tumor samples and matched normal blood were analyzed as pairs to detect somatic single nucleotide polymorphism (SNP) by MuTect2 (v4.0.5.1, https://gatk.broadinstitute.org/hc/en‐us/articles/360036730411‐Mutect2) and InDel by and Strelka2 (v2.8.3, https://github.com/Illumina/strelka).^[^ ^26^ ^]^ The VCF files of detected somatic mutations were annotated using Annovar.^[^ ^27^ ^]^ Since the false positive of somatic mutations is relatively high, in order to obtain high‐quality somatic mutations, a series of filters were carried out. The criteria are as follows: 1) The sequencing depth of tumor tissue and normal blood > = 10; 2) in the tumor samples, the number of reads supporting this variant > = 3; 3) in the tumor samples, the allele frequency of this variant > = 0.05; 4) in the normal blood, the allele frequency of this variant < = 0.01; 5) in the panel constructed by 80 normal people of the company (Genergy Biotechnology Co. Ltd.), the frequency of this variant in the panel < 0.05; or the frequency in the panel > = 0.05 and the allele frequency > = 0.30. The filtered somatic annotations were used for the subsequent analysis.

### Tumor Mutational Burden (TMB) Analysis

TMB is defined as the total number of somatic mutations per coding area of a tumor genome. The total number of single nucleotide substitution and InDel mutations per megabase in the coding region was counted in this study.

### Mutational Signature Analysis

Based on the location of somatic SNV, its anterior and posterior bases are found in the genome. These three bases form a three‐base mutational or somatic motifs. According to the specific somatic SNV and its three‐base mutation pattern of each sample, there will theoretically be 96 mutation motif distributions. Non‐negative matrix factorization (NMF) was used to compare the mutation characteristics decomposed by the mutation spectrum with the known features in the COSMIC database (v2), and look for similar mutation patterns in the database to interpret the mutation signatures of each sample with R package deconstructSigs (http://cancer.sanger.ac.uk/cosmic/signatures).

### RNA‐seq Data Analysis—RNA‐seq Data Processing and Quality Control

Trimmomatic (v0.39, http://www.usadellab.org/cms/index.php?page = trimmomatic) was used to remove adapter sequence fragments and low‐quality fragments from the sequencing data. FastQC (v0.11.5, http://www.bioinformatics.babraham.ac.uk/projects/fastqc) was used to perform quality control analysis on the preprocessed data. For each sample, the pretreated sequence was compared with the human reference genome (hg38) using STAR (2.7.3a, https://github.com/alexdobin/STAR).

### RNA‐seq Data Analysis—Quantification of Mutated Gene Expression

The input FASTQ files of RNA‐seq were scanned by MutScan (v1.14.0) to grab all the reads of the somatic mutation sites detected from WGS.^[^ ^28^ ^]^ Transcript quantification was performed using RSEM (v1.3.3, http://deweylab.github.io/RSEM). The expression value of the neoantigen‐associated mutated gene was calculated by

(the number of mutated reads)/(the number of total reads) ∗ (the expression value of the corresponding gene).

### RNA‐seq Data Analysis—Single Cell RNA‐seq Analysis

The preprocessed data were analyzed for quality control by FastQC (v0.11.5). Data quality statistics were performed on the raw FASTQ files using 10X in‐house software Cell Ranger. The comparison to the hg19 human reference genome and quantification of single‐cell transcriptomes were also performed on the FASTQ files. Samtools (v1.9) in VariantAnalysis was used to do SNP and InDel calling with bam file from CellRanger.^[^ ^29^ ^]^ In order to get high quality SNP and InDel sets, the filtering criteria of Samtools were set as follows: QUAL mass fraction > 10, base coverage depth DP > 20, SnpGap = 5, and IndelGap = 5. Annovar was used to annotate the variant sets. The organoid cells were clustered by Seurat (v4.0.2, https://satijalab.org/seurat) and cells carrying neoantigen‐associated mutations were labeled according to the barcode information of the cells.

### HLA Typing

4‐digit HLA‐class‐I alleles (HLA‐A, B, and ‐C) of tissues, organoids and patients’ PBMCs were analyzed with Athlates,^[^ ^30^ ^]^ HLAHD,^[^ ^31^ ^]^ and HLAVB^[^ ^32^ ^]^ using WGS data (Genergy Biotechnology, Shanghai, China). The overlapped results of the above three software were used as the HLA‐class‐I alleles of each sample for the subsequent analysis. The HLA‐class‐I alleles of five organoids used in peptide tests and healthy PBMCs were identified by PCR‐sequence‐based typing (PCR‐SBT) (CSTB, Shanghai, China). HLA matched healthy PBMCs were used in the study (Table S2, Supporting Information).

### Neoantigen Peptide Prediction and Peptide Synthesis

Based on the annotated VCF files, peptides were constructed by sliding a window over the affected mutated positions. For each nonsynonymous SNV and nonframeshift InDel shared by paired tissues and organoids, 9–11mer peptides containing the mutated amino acid to HLA‐A, ‐B, and ‐C alleles were predicted with Score tools (https://github.com/bm2‐lab/neoFusion).^[^ ^9^ ^]^ The detailed algorithms were described in supplementary method. Neoantigen peptides binding to the HLA alleles shared by tissues, organoids and patients’ PBMCs were predicted and scored. In each case, predicted peptides with the top high scores were selected for the validation experiment (Tables S2 and S3, Supporting Information). Customized peptides were obtained from GL Biochem (Shanghai, China) and dissolved in DMSO for experiments.

### HLA Class I Peptide Purification by Coimmunoprecipitation (CO‐IP) and Mass Spectrometry Analysis

Single cells of PDOs were washed twice with PBS. Pellet of PDOs (1–2 × 10^7^ cells pellet^−1^) were lysed in cold Western and IP lysis buffer containing Protease Inhibitors Cocktail (Beyotime) with shake on ice for 40 min. Cell lysates were cleared by centrifugation at 4 ℃ at 12 000 rpm for 10 min. Soluble lysates were coincubated with anti‐HLA class I antibody (W6/32 Abcam) and Protein A/G Magnetic Beads (MCE) with rotation for 2 h at 4 °C according to the manufacturer's instructions. After the beads were washed and eluted, supernatant was denatured and prepared for 10% sodium dodecyl sulfate‐polyacrylamide gel (SDS‐PAG). The collected stacking gel pieces were incubated with 50 µL dithiothreitol (DTT) solution (10 × 10^−3^ m) at 56 ℃ for 30 min and 50 µL iodoacetamide solution (55 × 10^−3^ m) at 37 ℃ in dark for 10 min, then neat acetonitrile was added to shrink the gel pieces. After the liquid was completely removed, the gel pieces were incubated with trypsin buffer (13 ng µL^−1^, in 10 × 10^−3^ m NH4HCO3 containing 10% acetonitrile) at 37 ℃ overnight. The digested peptides were extracted from the gel by sequentially adding 0.1% formic acid in 50% acetonitrile, 0.1% formic acid in 80% acetonitrile, and 100% acetonitrile.

The samples were analyzed by on‐line nanospray LC‐MS/MS on Q Exactive Plus mass spectrometer coupled to an EASY‐nanoLC 1000 system (Thermo Fisher Scientific; Kigene, Shanghai, China). 4 µL peptides was loaded (analytical column: Acclaim PepMap C18, 75 µm × 25 cm) in chromatographic gradient buffer (A: 0.1% formic acid in water, B: 0.1% formic acid in CAN) at a flow rate of 300 nL min^−1^ and separated with a 60 min gradient. Tandem mass spectra were processed by PEAKS Studio version X+ (Bioinformatics Solutions Inc.). PEAKS DB was set up to search the uniprot_Homo_sapiens (version201907, 20 414 entries) and the database of neoantigen peptides predicted.

Two predicted neoantigen peptides of organoids (HCC 25, HCC 448) and one predicted neoantigen peptide of tumor tissue (HCC 217) were detected with MS and selected for the validation experiment (Table S3 and Figure S5, Supporting Information).

### In Vitro Culture with Peptide

Healthy PBMCs were used to evaluate the immunogenicity of candidate neoantigen peptides. At least three HLA matched healthy peripheral blood (HPB) for each HCC were analyzed. PBMCs were cultured with 25 µL mL^−1^ ImmunoCult Human CD3/CD28 T Cell Activator in ImmunoCult‐XF T Cell Expansion Medium supplemented with 10 ng mL^−1^ Human Recombinant IL‐2 (STEMCELL Technologies) at 1 × 10^6^ cells mL^−1^ at 37 ℃ 5% CO_2_ for 3–6 days by adding fresh medium every 3 days for initial nonspecific expansion. 96 well U‐bottom were coated with 5 µg mL^−1^ anti‐CD28 (clone 28.2, ebioscience) at 4 ℃ overnight before use. On day 0, 1 × 10^5^ PBMCs were incubated with 25 × 10^−6^ m peptide (since customized peptides were dissolved in DMSO, PBMCs treated with DMSO were used as negative controls) in 200 µL T cell medium (AIM‐V medium supplemented with 10% human AB serum (Gemini), 1% Ultraglutamine, 1% penicillin/streptomycin, IL‐2 (100 U mL^−1^ PeproTech), IL‐7 (10 ng mL^−1^ PeproTech), IL‐15 (10 ng mL^−1^ PeproTech)) in each well with at least two replicates. On day 3, the cells were evenly split and 100 µL T cell medium (2× concentrated) was added. On day 6, half of the medium including peptide and cytokines (2× concentrated) was refreshed. Cell culture supernatants were completely gathered for enzyme‐linked immunosorbent assay (ELISA) analysis. To analyze the activity of ICIs, 1 × 10^5^ PBMCs per well were stimulated with peptides derived from the same HLA allele (25 × 10^−6^ m each peptide, in HCC 277 HLA‐A binding peptides were divided into two pools (peptide 1–5 and peptide 6–10, respectively)) for three cycles, 5 µg mL^−1^ PD‐1 inhibitor (Selleckchem) and/or 10 µg mL^−1^ CTLA‐4 inhibitor (Selleckchem) was added. Half of the medium including ICIs (2× concentrated) was refreshed at 3‐day intervals. Each peptide or peptide pool stimulation was repeated twice. After three cycles of peptide stimulation, PBMCs were collected and T cell response markers was assessed by flow cytometry.

### T Cell Response Analysis by Flow Cytometry

PBMCs stimulated with the same peptides were collected together, washed twice, and stained with FV450 (BD) for 15 min at room temperature (RT) and anti CD107a PE‐Cy7, anti CD3 FITC, anti CD45 APC‐Cy7, anti CD4 PE, anti CD8 APC, and anti CD137 PE‐Cy5 (BD) for 30 min at 4 ℃. Cells were washed twice in FACS buffer, fixed and permed using Fixation/Permeabilization kit (PeproTech), and stained for intracellular IFN‐*γ* (anti IFN‐*γ* BV510, Biolegend) for 15 min at 4 ℃. Cells were washed twice with FACS buffer before analyzed with Canto II (BD) or CytoFLEX (Beckman) flow cytometer.

### IFN‐*γ* ELISA Assay

IFN‐*γ* in supernatants was quantified with human IFN‐*γ* High sensitivity ELISA kit (Abcam) according to the manufacturer's instructions.

### Organoid Killing Assay

PBMCs were incubated with immunogenic peptide pools for nine days with three cycles of stimulations. After stimulation, PBMCs were washed and stained with anti CD3 AF700, anti CD8 APC, and Propidium iodide (PI, BD). Peptide specific live T cells (CD3^+^CD8^+^PI^−^) were FACS isolated for organoid killing assay. Organoids were dissociated to single cells with Tryple Express, labeled with CFSE (BD), and cocultured in at least triplicate with sorted T cells at a 10:1 effector:target ratio in T cell culture medium. To analyze the activity of ICIs, 5 µg mL^−1^ PD‐1 inhibitor and/or 10 µg mL^−1^ CTLA‐4 inhibitor was added during the progress of stimulation and killing assay. Each organoid killing assay was repeated twice. After 3 days of coculture, organoids and T cells were collected. Cells were washed in FACS buffer and stained with anti CD45 APC‐Cy7, Annexin V PE, 7‐AAD (BD) in Annexin V buffer (BD) for 15 min at RT. The apoptosis of organoids labeled with CFSE were analyzed with flow cytometer Canto II (BD) or CytoFLEX (Beckman). FITC^+^CD45 APC‐Cy7^−^ was used to detect organoids and Annexin V^−^7‐AAD^−^ was used to define live cells.

### Statistical Analysis

The data set here consisted of 27 pairs of tissues and organoids in sequencing analysis, five organoids and 16 PB from healthy donors in validation experiment. Statistical analysis was performed using SPSS Statistics 23 (IBM). GraphPad Prism 9 and Adobe Illustrator 2021 were used for figure presentation. In flow cytometry and ELISA assays, the results of experimental groups were calculated by the fold change. In organoid killing assay, the results were calculated by the differences between experimental groups and control groups. All values are presented as the mean ± standard deviation (SD). For statistical evaluation, two‐tailed paired or unpaired *t*‐test was performed to compare paired or unpaired groups, Spearman's Rho was used to analyze the correlation between groups, multiple groups were compared with ANOVA followed by post‐hoc test (LSD), Pearson's chi‐squared test was used to compare the enrichment of subgroups. In all cases, *P* < 0.05 was considered as statistically significant difference.

## Conflict of Interest

The authors declare no conflict of interest.

## Author Contributions

W.W., T.Y., L.M., and Y.Z. contributed equally to this work. L.C. and P.W. designed experiments. H.Y.W., D.G., P.W., L.C., W.W.W., T.G.Y., L.L.M., and Y.J.Z. developed methods. Y.J.Z., X.F.Z., Y.Z., S.Y., X.Y.Q., S.Y.S., R.W., and T.W. collected samples. W.W.W., T.G.Y., Y.J.Z., J.X.B., Y.L.Z., and Y.N.Z. performed experiments. W.W.W., T.G.Y., L.L.M., and Y.J.Z. analyzed data. W.W.W. and T.G.Y. wrote the manuscript, all authors revised the manuscript and approved the final version. L.C., P.W., H.Y.W., and D.G. supervised the project.

## Supporting information

Supporting Information

## Data Availability

The data that support the findings of this study are available from the corresponding author upon reasonable request.
