# Supplementary material for: Hepatobiliary Tumor Organoids Reveal HLA Class I Neoantigen Landscape and Antitumoral Activity of Neoantigen Peptide Enhanced with Immune Checkpoint Inhibitors
Source: Adv Sci (Weinh). 2022 Jun 5;9(22):2105810. doi: 10.1002/advs.202105810 (PMC9353440; doi:10.1002/advs.202105810)
Supplement: Supplementary file 1 — Supporting Information [file ADVS-9-2105810-s001.pdf]

Hepatobiliary Tumor Organoids Reveal HLA Class I Neoantigen Landscape and Antitumoral Activity of Neoantigen Peptide Enhanced with Immune Checkpoint Inhibitors

Authors

Wenwen Wang[1,†], Tinggan Yuan[2,3,4,†], Lili Ma[2,3,4,†], Yanjing Zhu[5,6,†], Jinxia Bao[7], Xiaofang Zhao[1], Yan Zhao[8], Yali Zong[8], Yani Zhang[8], Shuai Yang[1], Xinyao Qiu[1], Siyun Shen[5,6], Rui Wu[9], Tong Wu[5,6], Hongyang Wang[1,5,6,*], Dong Gao[4,10,11,*], Peng Wang[2,3,4,*], Lei Chen[1,6,12,13,*]

Supplementary Materials

- **Supplementary method**
- **Figure S1.** Organoids maintain genetic landscape of matched tissues.
- **Figure S2.** Tumor purity of tissues and matched organoids.
- **Figure S3.** Relationships between TMB/neoantigen/HSN and clinical characteristics in hepatobiliary cancer.
- **Figure S4.** Neoantigen peptide related features and neoantigen-associated mutation related SNV types in tissues and organoids.
- **Figure S5.** HLA-A, -B, and -C binding peptides detected with mass spectrometry.
- **Figure S6.** Immunogenicity of peptides judged by $CD107a^{+}IFN\gamma^{+}$ expressions of peptide-reactive T cells.
- **Figure S7.** Bright field images of organoids for validation experiment.
- **Figure S8.** Predicted scores of peptide presentation features of 9mer and 11mer length peptides in tissues and organoids.
- **Figure S9.** The distribution of predicted neoantigen-associated mutation analyzed at single cell level.
- **Table S1.** HLA-A, -B, and -C information of PBMCs, tissues, organoids and clinical characteristics of 27 patients.
- **Table S2.** HLA-A, -B, and -C information of healthy PBMCs.
- **Table S3.** Selected peptides for validation experiment in five patients.
- **Table S4.** Features of immunogenic peptides in validation experiment.
- **Table S5.** Immunogenic potential of peptides with different length and HLA allele
- **Supplementary reference**

Supplementary method

Immunogenic Potential Score

Score tools previously published were used here to quantitatively assess the immunogenic potential of peptides.[1] These tools integrate multiple approaches to predicting the immunogenicity of epitopes at different stages of antigen presentation, including HLA binding affinity (NetMHCpan4.1/4.0, *Rm*: the binding affinity percent rank of mutated peptide, *Rn*: the binding affinity percent rank of the corresponding wild type peptide, *m*: mismatch between mutated and the corresponding wild type peptide), the likelihood of pMHC recognized by T-cell receptors (fitness model, *H*: the probability of pMHC recognized by T cells, *R*: the likelihood of a neoantigen recognized by the TCR repertoire), TAP transport efficiency and proteasomal cleavage (NetCTLpan 1.1, *C*: Combined score of binding affinity, proteasomal C' terminal cleavage, and TAP transport efficiency). For early peptide analysis of HCC 33 and HCC 277, NetMHCpan 4.0 was used for peptide scoring; in other analysis, NetMHCpan 4.1 was used.

The score to assess the immunogenic potential of predicted peptides was defined as follows:

$A = C * L(Rm)$

$B = H * R * (1 - 2^{-m} L(Rn))$

$L(x) = 1/(1+e^{5(x-2)})$ (*L(x)* is a negative logistic function)

$Score = A * B.$

Tumor Purity Analysis

Tumor purity was inferred by Sequenza for whole genome sequencing (WGS) data;[2] cell malignancy of single cell RNA-seq data was estimated with R package "scCancer"[3] which calculated the malignancy scores based on infercnv algorithm (https://github.com/broadinstitute/inferCNV).

Supplemental Figures

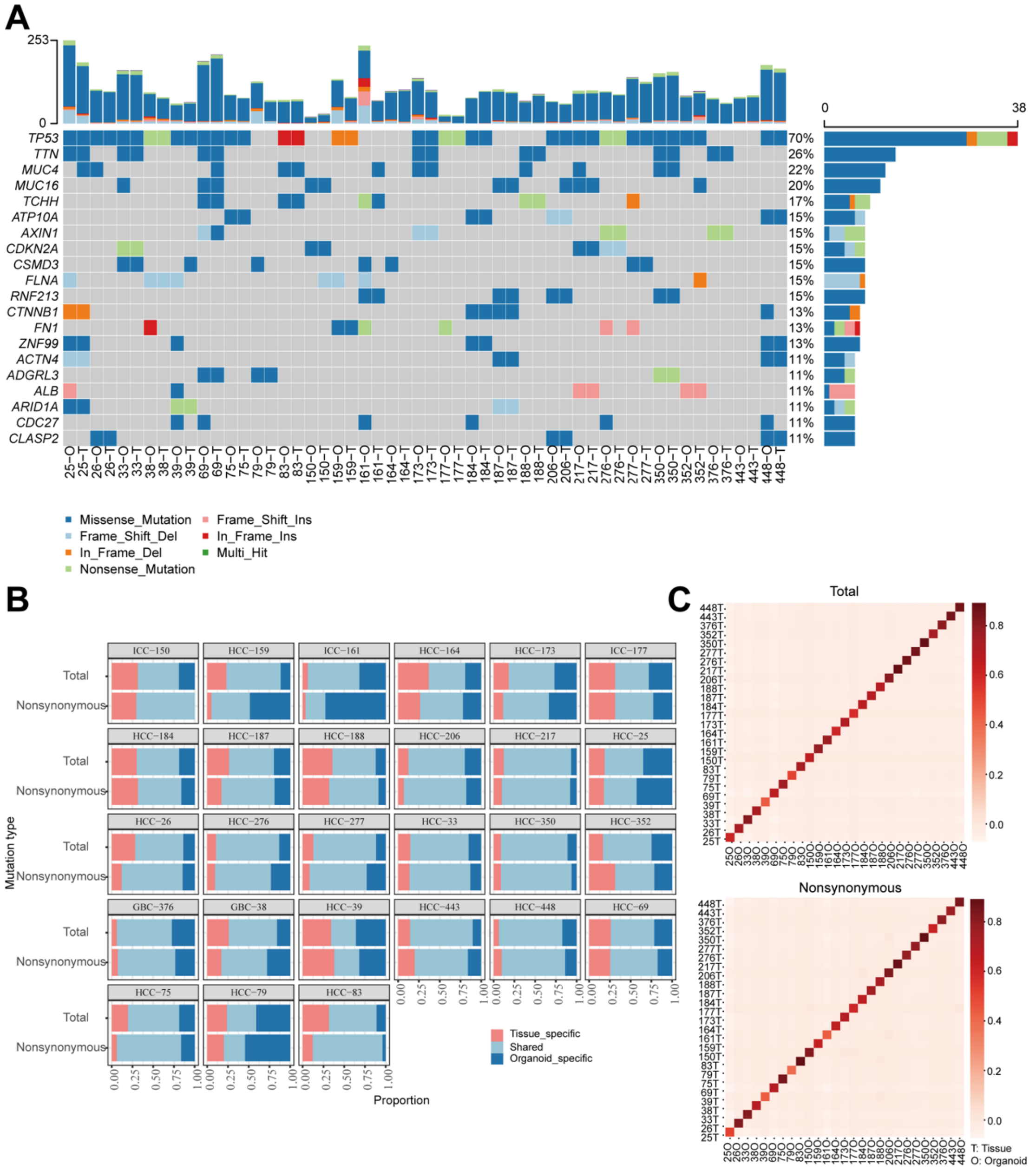

Fig. S1. Organoids maintain genetic landscape of matched tissues.

A) Data of top 20 high-frequency mutations of paired tissues and organoids for all 27 patients with hepatobiliary cancer. T: tissue, O: organoid.

B) Proportions of total and nonsynonymous mutations detected only in tissues (red), only in organoids (dark blue) or shared (light blue) within each patient.

C) Corregram indicating total and nonsynonymous mutations of each organoid correlated to its corresponding tissue, but rarely with others. Color key from dark brown to light brown indicates correlation from high to low.

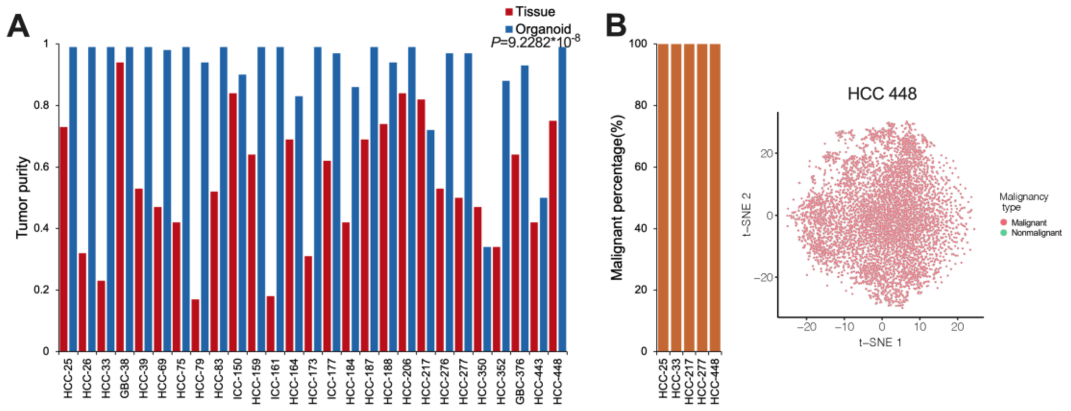

Fig. S2. Tumor purity of tissues and matched organoids.

A) Tumor purity of paired tissues and organoids for all 27 patients with hepatobiliary cancer. *P*-value was calculated by two-tailed paired t test.

B) Percentages of malignant single cells for five organoids. Representative t-SNE plot of all single cells for HCC 448 colored by malignant (red) and nonmalignant (green) cells.

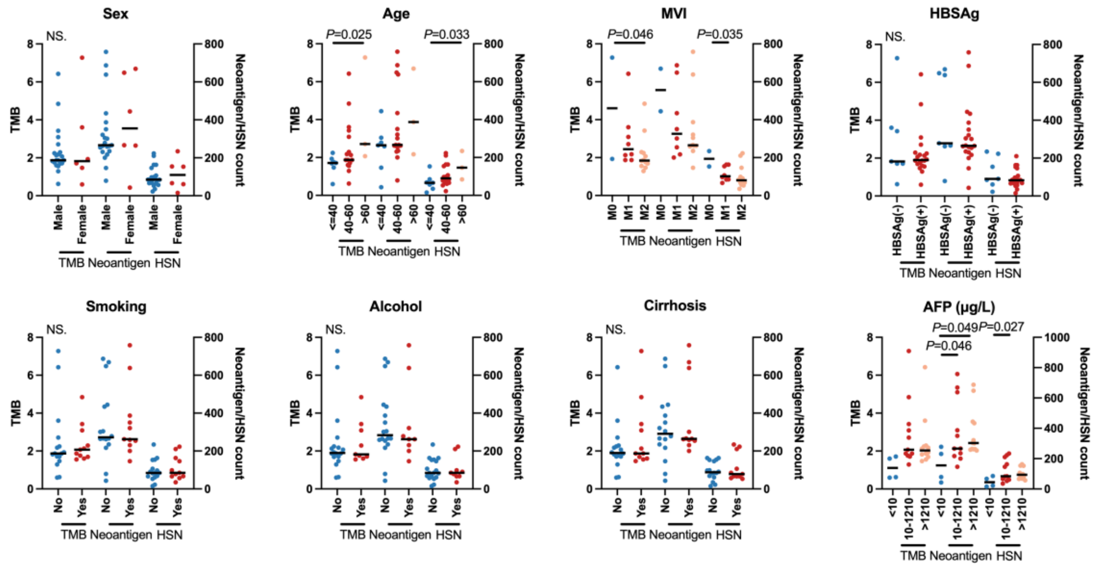

Fig. S3. Relationships between TMB/neoantigen/HSN and clinical characteristics in hepatobiliary cancer.

TMB and neoantigen/high score neoantigen peptide (HSN) load of groups divided by sex, age, microvascular invasion (MVI), HBSAg, smoking, alcohol, cirrhosis status and serum AFP value were compared (n=27). *P*-values were calculated with ANOVA followed by post-hoc test (LSD). NS.: not significant.

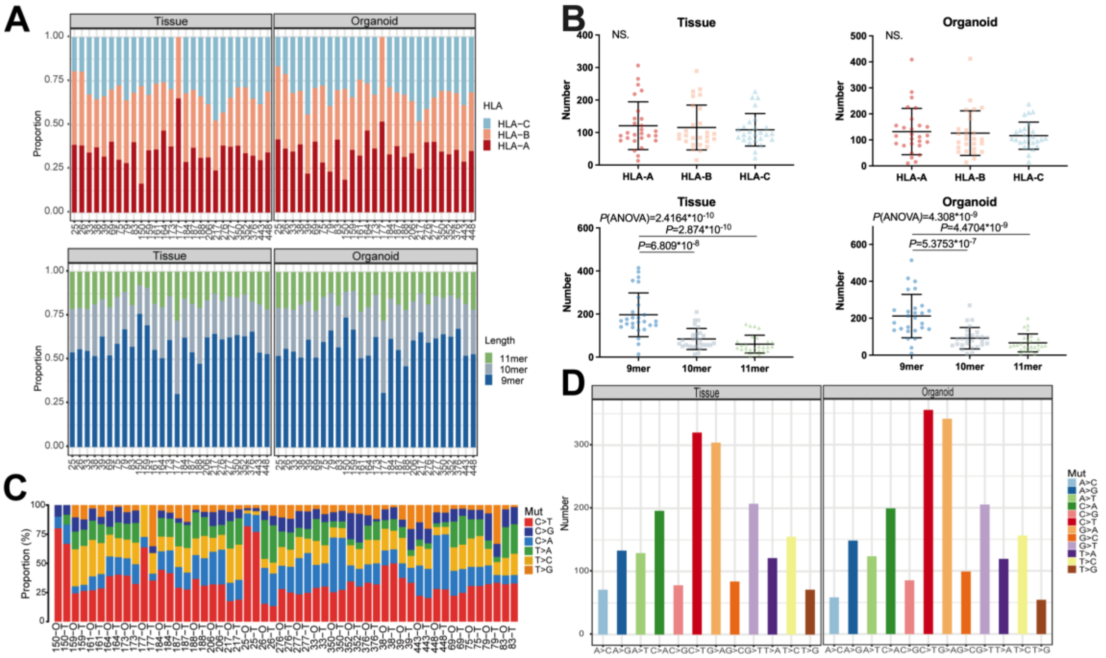

Fig. S4. Neoantigen peptide related features and neoantigen-associated mutation related SNV types in tissues and organoids.

A) Proportions of predicted neoantigen peptides with each HLA allele and each length for individual tissues and organoids.

B) Numbers of predicted neoantigen peptides with each HLA allele and each length for individual tissues and organoids (n=27). Data are presented as mean ± SD. *P*-values were calculated with ANOVA followed by post-hoc test (LSD). NS.: not significant.

C) Proportions of six neoantigen-associated mutation related SNV types of paired tissues and organoids for 27 patients with hepatobiliary cancer. T: tissue, O: organoid.

D) Numbers of 12 neoantigen-associated mutation related SNV types in tissues and organoids (n=27 pairs).

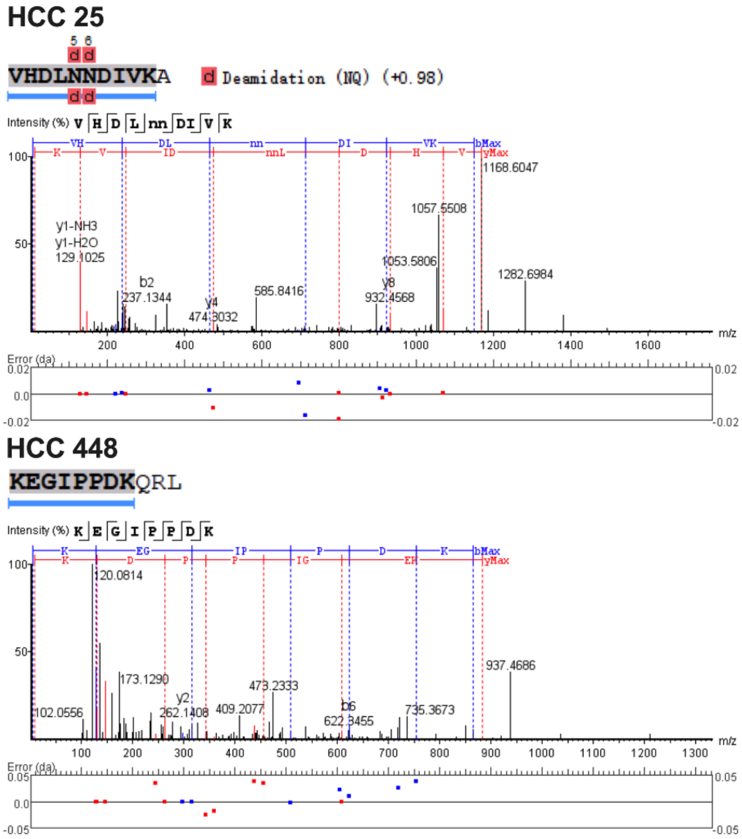

Fig. S5. HLA-A, -B, and -C binding peptides detected with mass spectrometry. Neoantigen associated-mutation derived HLA-A, -B, and -C-presented peptides of organoids confirmed with mass spectrometry for validation experiments.

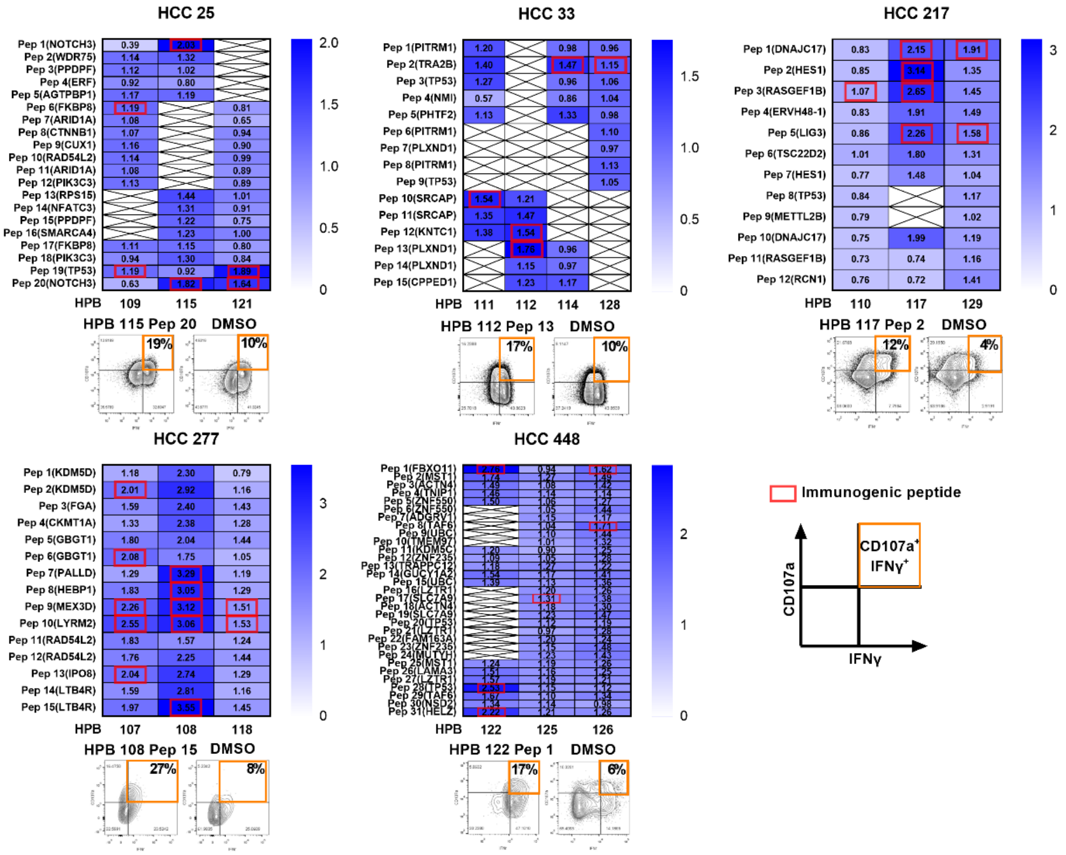

Fig. S6. Immunogenicity of peptides judged by CD107a^{+}IFNγ^{+} expressions of peptide-reactive T cells.

DMSO stimulation was used as control and the detailed fold changes of CD107a^{+}IFNγ^{+} co-expression of CD3^{+}CD8^{+} T cells after three cycles of each peptide stimulation in five HCC organoids were shown. Representative flow cytometry plots gated on CD45^{+}CD3^{+}CD8^{+} live T cells.

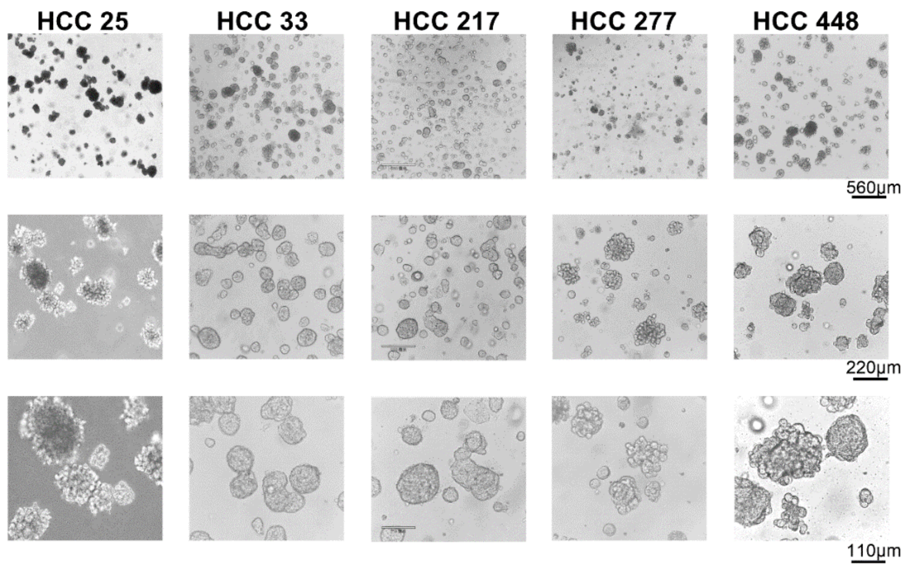

Fig. S7. Bright field images of organoids for validation experiment.

Microscopic appearance of hepatobiliary tumor tissue-derived organoids. Scale bars of each row were shown.

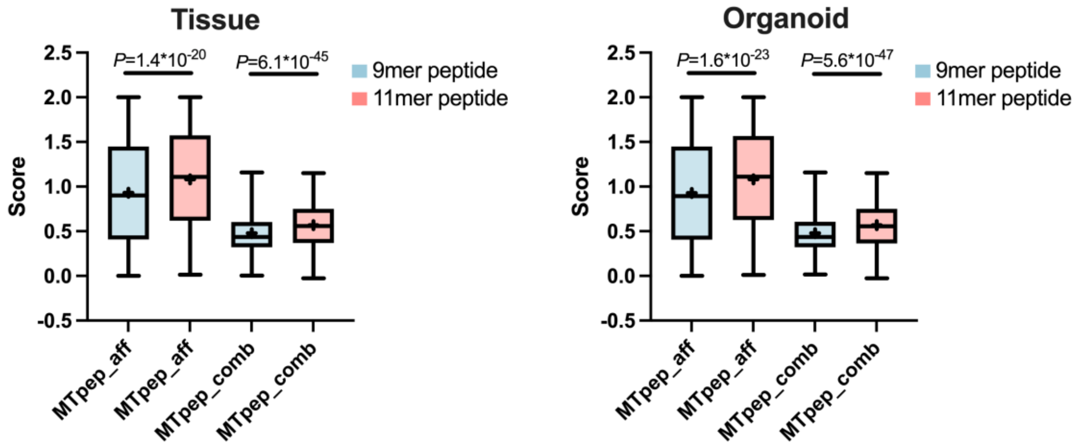

Fig. S8. Predicted scores of peptide presentation features of 9mer and 11mer length peptides in tissues and organoids.

The scores of mutated peptide binding affinity, proteasomal C' terminal cleavage and TAP transport combined efficiency of 9mer and 11mer peptides in tissues and paired organoids were compared (n=27). Mean was shown as '+'. *P*-values were calculated with two-tailed unpaired t test.

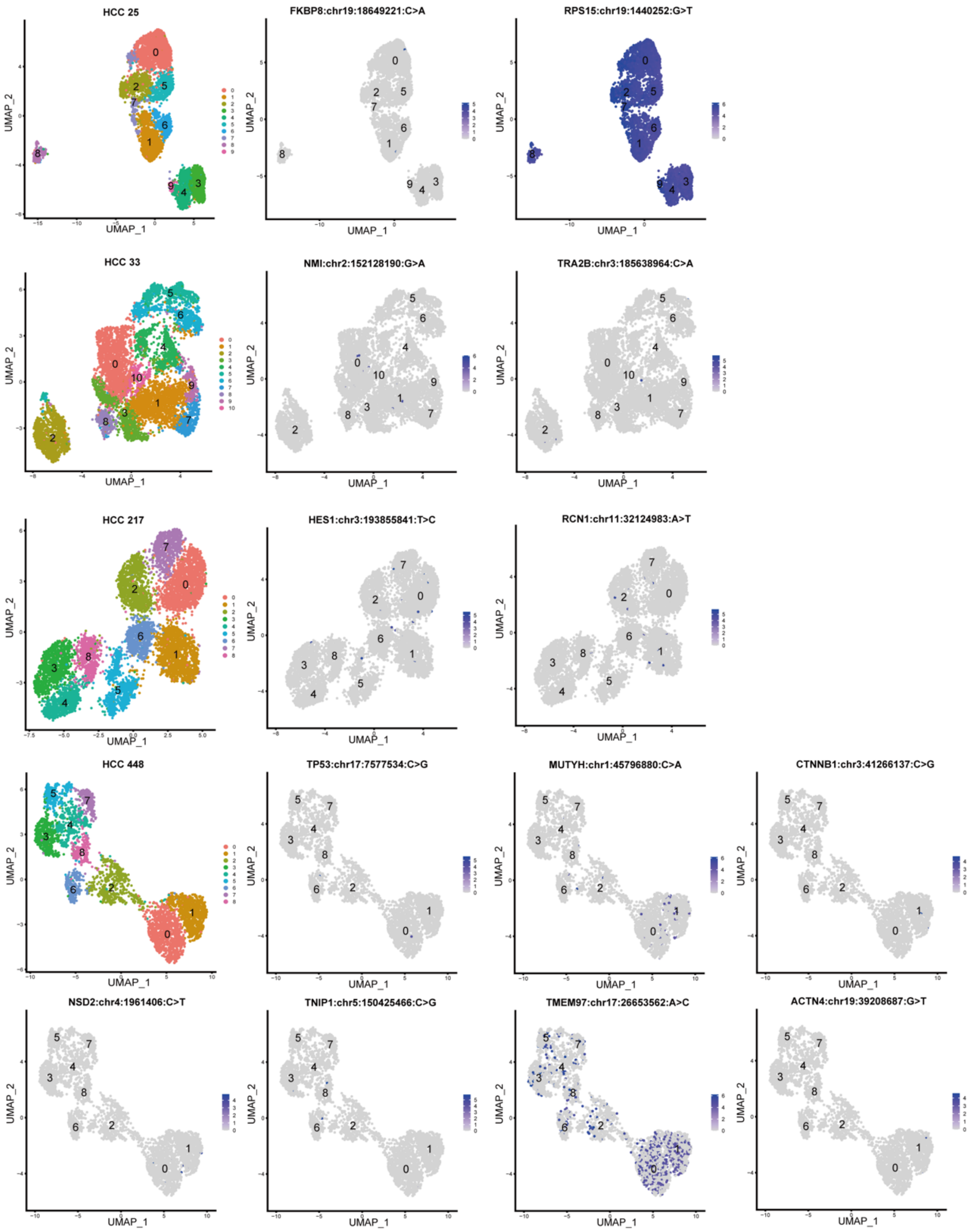

Fig. S9. The distribution of predicted neoantigen-associated mutation analyzed at single cell level.

UMAP plot of all single cells for HCC 25, HCC 33, HCC 217, HCC 448 reveals different clusters. UMAP plot of all single cells colored by different neoantigen-associated mutations.

Supplemental Tables

Table S1. HLA-A, -B, and -C information of PBMCs, tissues, organoids and clinical characteristics of 27 patients

Patient No.	Sample type	HLA-A		HLA-B		HLA-C		Sex	Age	MVI	Tumor stage	Pathology	HBsAg	Smoking	Alcohol	Cirrhosis	AFP(μg/L)	HBV infection
25	PB	A*24:02	A*02:01	B*40:03	B*40:01	C*03:04	C*03:04	Male	60	M1	II	HCC	+	No	No	No	>1210	Yes
	Tissue	A*24:02	A*02:01	B*40:03	B*40:01	C*03:04	C*03:04											
	Organoid	A*24:02	A*02:01	B*40:03	B*40:01	C*03:04	C*03:04											
26	PB	A*02:01	A*11:01	B*38:02	B*39:01	C*07:02	C*07:02	Male	45	M1	II	HCC	+	No	No	No	>1210	Yes
	Tissue	A*02:01	A*11:01	B*38:02	B*39:01	C*07:02	C*07:02											
	Organoid	A*02:01	A*11:01	B*38:02	B*39:01	C*07:02	C*07:02											
33	PB	A*32:01	A*01:01	B*07:02	B*57:01	C*07:02	C*06:02	Male	56	M2	II	HCC	-	Yes	Yes	Yes	101.7	Yes
	Tissue	A*32:01	A*01:01	B*07:02	B*57:01	C*07:02	C*06:02											
	Organoid	A*32:01	A*01:01	B*07:02	B*57:01	C*07:02	C*06:02											
38	PB	A*31:01	A*11:01	B*15:01	B*40:02	C*03:04	C*04:01	Female	55	NA	IVB	GBC	-	No	No	No	233.8	No
	Tissue	A*31:01	A*11:01	B*15:01	B*40:02	C*03:04	C*04:01											
	Organoid	A*31:01	A*11:01	B*15:01	B*40:02	C*03:04	C*04:01											
39	PB	A*02:06	A*02:07	B*46:01	B*57:01	C*01:02	C*06:02	Male	48	M2	II	HCC	+	No	No	No	447	Yes
	Tissue	A*02:06	A*02:07	B*46:01	B*57:01	C*01:02	C*06:02											
	Organoid	A*02:06	A*02:07	B*46:01	B*57:01	C*01:02	C*06:02											
69	PB	A*33:03	A*11:01	B*44:03	B*15:02	C*14:03	C*08:01	Male	54	M2	IIIA	HCC	+	Yes	Yes	Yes	479.2	Yes
	Tissue	A*33:03	A*11:01	B*44:03	B*15:02	C*14:03	C*08:01											
	Organoid	A*33:03	A*11:01	B*44:03	B*15:02	C*14:03	C*08:01											
75	PB	A*24:02	A*24:02	B*15:27	B*40:02	C*04:01	C*03:04	Male	39	M2	IIIB	HCC	+	Yes	Yes	No	75.4	Yes
	Tissue	A*24:02	A*24:02	B*15:27	B*40:02	C*04:01	C*03:04											
	Organoid	A*24:02	A*24:02	B*15:27	B*15:27	C*04:01	C*04:01											
79	PB	A*02:03	A*02:07	B*55:02	B*46:01	C*01:02	C*12:03	Male	58	M2	IIIB	HCC	+	Yes	Yes	Yes	>1210	Yes
	Tissue	A*02:03	A*02:07	B*55:02	B*46:01	C*01:02	C*12:03											
	Organoid	A*02:03	A*02:07	B*55:02	B*46:01	C*01:02	C*12:03											
83	PB	A*68:01	A*11:01	B*52:01	B*40:01	C*07:02	C*12:02	Male	35	M2	IVA	HCC	+	Yes	No	Yes	>1210	Yes
	Tissue	A*68:01	A*11:01	B*52:01	B*40:01	C*07:02	C*12:02											
	Organoid	A*68:01	A*11:01	B*52:01	B*40:01	C*07:02	C*12:02											
150	PB	A*30:01	A*30:01	B*13:02	B*13:01	C*06:02	C*06:02	Male	49	NA	I	ICC	-	No	No	No	2.5	No
	Tissue	A*30:01	A*30:01	B*13:02	B*13:01	C*06:02	C*06:02											
	Organoid	A*30:01	A*30:01	B*13:02	B*13:01	C*06:02	C*06:02											
159	PB	A*02:07	A*30:01	B*46:01	B*13:02	C*01:03	C*06:02	Male	55	M2	IIIB	HCC	-	No	Yes	No	>1210	Yes
	Tissue	A*02:07	A*30:01	B*46:01	B*13:02	C*01:03	C*06:02											
	Organoid	A*02:07	A*30:01	B*46:01	B*13:02	C*01:03	C*06:02											
161	PB	A*02:06	A*33:03	B*44:03	B*40:06	C*08:01	C*14:03	Male	58	NA	I	ICC	+	Yes	Yes	Yes	9.9	Yes
	Tissue	A*02:06	A*33:03	B*44:03	B*40:06	C*08:01	C*14:03											
	Organoid	A*02:06	A*33:03	B*44:03	B*40:06	C*08:01	C*14:03											
164	PB	A*11:01	A*11:02	B*46:01	B*46:01	C*01:02	C*01:02	Male	36	M2	II	HCC	+	No	No	No	>1210	No
	Tissue	A*11:01	A*11:02	B*46:01	B*46:01	C*01:02	C*01:02											
	Organoid	A*11:01	A*11:02	B*46:01	B*46:01	C*01:02	C*01:02											
173	PB	A*02:01	A*02:03	B*46:01	B*13:01	C*15:02	C*03:04	Male	42	M2	IIIB	HCC	+	Yes	Yes	No	>1210	Yes
	Tissue	A*02:01	A*02:03	B*46:01	B*13:01	C*15:02	C*03:04											
	Organoid	A*02:01	A*02:03	B*46:01	B*13:01	C*15:02	C*03:04											
177	PB	A*11:01	A*24:02	B*15:01	B*15:32	C*01:02	C*12:02	Female	32	NA	IIIB	ICC	+	No	No	No	5.8	Yes
	Tissue	A*11:01	A*11:01	B*15:32	B*15:32	C*12:03	C*12:03											
	Organoid	A*11:01	A*11:01	B*15:32	B*15:32	C*12:03	C*12:03											
184	PB	A*11:02	A*11:01	B*13:01	B*38:02	C*07:02	C*03:04	Female	38	M0	I	HCC	+	No	No	No	>1210	Yes
	Tissue	A*11:02	A*11:01	B*13:01	B*38:02	C*07:02	C*03:04											
	Organoid	A*11:02	A*11:01	B*13:01	B*38:02	C*07:02	C*03:04											
187	PB	A*02:01	A*11:02	B*27:04	B*40:01	C*07:02	C*12:02	Male	62	M2	IIIB	HCC	+	Yes	No	No	12.2	Yes
	Tissue	A*02:01	A*11:02	B*27:04	B*40:01	C*07:02	C*12:02											
	Organoid	A*02:01	A*11:02	B*27:04	B*40:01	C*07:02	C*12:02											
188	PB	A*33:03	A*11:01	B*58:01	B*07:02	C*03:02	C*07:02	Male	46	M1	II	HCC	+	Yes	No	No	10.5	No
	Tissue	A*33:03	A*11:01	B*58:01	B*07:02	C*03:02	C*07:02											
	Organoid	A*33:03	A*11:01	B*58:01	B*07:02	C*03:02	C*07:02											
206	PB	A*33:03	A*01:01	B*37:01	B*58:01	C*03:02	C*06:02	Female	35	M2	IIIA	HCC	+	No	No	Yes	>1210	Yes
	Tissue	A*33:03	A*01:01	B*37:01	B*58:01	C*03:02	C*06:02											
	Organoid	A*33:03	A*01:01	B*37:01	B*58:01	C*03:02	C*06:02											
217	PB	A*33:01	A*24:02	B*58:01	B*15:09	C*03:02	C*07:04	Male	61	M1	IIIB	HCC	+	No	No	No	29.5	Yes
	Tissue	A*33:03	A*24:02	B*58:01	B*15:18	C*03:02	C*07:04											
	Organoid	A*33:03	A*24:02	B*58:01	B*15:18	C*03:02	C*07:04											
276	PB	A*02:07	A*11:01	B*40:01	B*40:01	C*03:04	C*07:02	Male	49	M1	II	HCC	+	Yes	No	Yes	>1210	Yes
	Tissue	A*02:07	A*11:01	B*40:01	B*40:01	C*03:04	C*07:02											
	Organoid	A*02:07	A*11:01	B*40:01	B*40:01	C*03:04	C*07:02											
277	PB	A*11:01	A*24:02	B*15:02	B*40:01	C*08:01	C*03:04	Male	48	M1	II	HCC	+	Yes	Yes	Yes	1210	Yes
	Tissue	A*11:01	A*11:01	B*15:02	B*15:02	C*08:01	C*08:01											
	Organoid	A*11:01	A*11:01	B*15:02	B*15:02	C*08:01	C*08:01											
350	PB	A*11:01	A*30:01	B*38:02	B*13:02	C*07:02	C*06:02	Female	59	M1	II	HCC	-	No	No	No	>1210	No
	Tissue	A*11:01	A*30:01	B*38:02	B*13:02	C*07:02	C*06:02											
	Organoid	A*11:01	A*30:01	B*38:02	B*13:02	C*07:02	C*06:02											
352	PB	A*68:01	A*02:07	B*46:01	B*15:27	C*01:02	C*04:01	Male	52	M2	II	HCC	+	No	No	Yes	15.2	Yes
	Tissue	A*68:01	A*02:07	B*46:01	B*15:27	C*01:02	C*04:01											
	Organoid	A*68:01	A*02:07	B*46:01	B*15:27	C*01:02	C*04:01											
376	PB	A*02:01	A*30:01	B*13:02	B*58:01	C*03:02	C*06:02	Male	27	NA	II	GBC	-	No	Yes	No	2.2	No
	Tissue	A*02:01	A*30:01	B*13:02	B*58:01	C*03:02	C*06:02											
	Organoid	A*02:01	A*30:01	B*13:02	B*58:01	C*03:02	C*06:02											
443	PB	A*01:01	A*11:01	B*40:02	B*57:01	C*06:02	C*07:02	Male	51	M1	IIIA	HCC	+	No	No	Yes	>1210	Yes
	Tissue	A*01:01	A*11:01	B*40:02	B*57:01	C*06:02	C*07:02											
	Organoid	A*01:01	A*11:01	B*40:02	B*57:01	C*06:02	C*07:02											
448	PB	A*11:01	A*33:03	B*40:01	B*58:01	C*03:02	C*07:02	Female	65	M0	I	HCC	-	No	No	Yes	209	No
	Tissue	A*11:01	A*33:03	B*40:01	B*58:01	C*03:02	C*07:02											
	Organoid	A*11:01	A*33:03	B*40:01	B*58:01	C*03:02	C*07:02											

PB: peripheral blood; No.: number; NA: not available
HCC: hepatocellular carcinoma; ICC: intrahepatic cholangiocarcinoma; GBC: gallbladder carcinoma

Table S2. HLA-A, -B, and -C information of healthy PBMCs

Matched patient No.	Healthy PB No.	HLA-A		HLA-B		HLA-C	
HCC 25	HPB 109	A*02:01	A*24:02	B*40:01	B*15:01	C*03:04	C*03:04
	HPB 115	A*02:01	A*03:01	B*07:02	B*40:03	C*03:04	C*07:02
	HPB 121	A*24:02	A*24:02	B*40:01	B*40:03	C*03:04	C*07:02
HCC 33	HPB 111	A*01:01	A*24:02	B*07:02	B*35:03	C*12:03	C*07:02
	HPB 112	A*02:01	A*24:02	B*07:02	B*57:01	C*06:02	C*07:02
	HPB 114	A*01:01	A*24:02	B*40:02	B*57:01	C*06:02	C*03:04
	HPB 128	A*01:01	A*32:01	B*35:01	B*35:03	C*04:01	C*04:01
HCC 217	HPB 110	A*24:02	A*33:03	B*51:01	B*58:01	C*03:02	C*14:02
	HPB 117	A*24:02	A*11:01	B*40:01	B*58:01	C*03:02	C*03:04
	HPB 129	A*24:02	A*33:03	B*58:01	B*58:01	C*03:02	C*03:02
HCC 277	HPB 107	A*11:01	A*02:07	B*15:02	B*40:01	C*03:04	C*08:01
	HPB 108	A*11:01	A*24:02	B*15:02	B*40:01	C*03:04	C*08:01
	HPB 118	A*11:01	A*03:01	B*15:02	B*52:01	C*12:02	C*08:01
HCC 448	HPB 122	A*11:01	A*68:01	B*40:01	B*51:01	C*15:02	C*07:02
	HPB 125	A*11:01	A*33:03	B*40:01	B*58:01	C*03:02	C*07:02
	HPB 126	A*11:01	A*33:03	B*40:01	B*58:01	C*03:02	C*07:02

PB: peripheral blood

Table S3. Selected peptides for validation experiment in five patients

Patient No.	Pep No.	HLA type	MTpep	WTpep	Gene
25	1	HLA-A*02:01	ILVLGVMVAL	ILVLGVMVAR	NOTCH3
	2	HLA-A*02:01	HLHLYSCSL	HLQLYSCSL	WDR75
	3	HLA-A*02:01	AIPHPLPKA	AIRHPLPYA	PPDPF
	4	HLA-A*02:01	SLPHDPGVFRV	RLPHDPGVFRV	ERF
	5	HLA-A*02:01	VHDLNNDIVKA	VHGLNNDIVKA	**AGTPBP1**(1)
	6	HLA-A*24:02	RSPYIPPHSAL	RSPYIPPHAAL	*FKBP8**
	7	HLA-A*24:02	EYFRRCRIEIF	EYFRRCLIEIF	ARID1A
	8	HLA-A*24:02	SYLTTTAPSL	SGATTTAPSL	CTNNB1
	9	HLA-A*24:02	LQWQLMQSF	LQRQLMQSF	CUX1
	10	HLA-A*24:02	RYSQKKPCYI	RYGQKKPCYI	RAD54L2
	11	HLA-B*40:01	VEYFRRCRI	VEYFRRCLI	ARID1A
	12	HLA-B*40:01	EEALHYMQSL	EEAVHYMQSL	PIK3C3
	13	HLA-B*40:03	VEINPEMIGHY	VEIKPEMIGHY	*RPS15**
	14	HLA-B*40:03	SSQELPHIEKY	SAQELPHIEKY	NFATC3
	15	HLA-B*40:03	GEAIPHPLP	GEAIPHPPG	PPDPF
	16	HLA-B*40:03	YEEEFDLFMRM	HEEEFDLFMRM	**SMARCA4**(2)
	17	HLA-C*03:04	YIPPHSALCL	YIPPHAALCL	*FKBP8**
	18	HLA-C*03:04	EALHYMQSL	EAVHYMQSL	PIK3C3
	19	HLA-C*03:04	SPILTIITL	RPILTIITL	TP53
	20	HLA-C*03:04	LVLGVMVAL	LVLGVMVAR	NOTCH3
33	1	HLA-A*01:01	SNRACEWALQY	SNRACERALQY	PITRM1
	2	HLA-A*01:01	SLRRDYYDRGY	SRRRDYYDRGY	*TRA2B**
	3	HLA-A*01:01	NTFLHSVVVPY	NTFRHSVVVPY	TP53
	4	HLA-A*01:01	YPLYINQTCY	YPLYINQTCH	*NMI**
	5	HLA-A*01:01	MSVLEICGMIM	MSVLEISGMIM	PHTF2
	6	HLA-A*32:01	RACEWALQYKL	RACERALQYKL	PITRM1
	7	HLA-A*32:01	VVAADAYCGW	VGAADAYCGW	PLXND1
	8	HLA-A*32:01	RACEWALQY	RACERALQY	PITRM1
	9	HLA-A*32:01	FLHSVVVPY	FRHSVVVPY	TP53
	10	HLA-B*07:02	RPRPTPASTPA	RPRPTPASAPA	SRCAP
	11	HLA-B*07:02	RPTPASTPA	RPTPASAPA	SRCAP
	12	HLA-B*07:02	WPSLHIEEF	WPSLHVEEF	KNTC1
	13	HLA-B*57:01	CVVAADAYCGW	CVGAADAYCGW	PLXND1
	14	HLA-B*57:01	VAADAYCGW	GAADAYCGW	PLXND1
	15	HLA-B*57:01	ISAAEAGGVF	MSAAEAGGVF	CPPED1
217	1	HLA-A*24:02	GYSKDVLPRL	GYSKDVLLRL	DNAJC17
	2	HLA-A*24:02	AVPAPDGQFAF	VVPAPDGQFAF	*HES1**
	3	HLA-A*24:02	FFGLLIKDIYF	FFSLLIKDIYF	RASGEF1B
	4	HLA-B*58:01	NGAICPKGKQW	NGAIIVSGKQK	ERVH48-1
	5	HLA-B*58:01	MGCYDPGRQKW	MGCYDPGSQKW	LIG3
	6	HLA-B*58:01	KSEPLPQPSL	KSEPLPQPPL	TSC22D2
	7	HLA-B*58:01	QAVPAPDGQF	QVVPAPDGQF	*HES1**
	8	HLA-A*33:03	CSCMGGMNR	SSCMGGMNR	TP53
	9	HLA-A*33:03	DWFLENKSEVR	DWFLENKSEVC	**METTL2B**(3)
	10	HLA-C*03:02	YSKDVLPRL	YSKDVLLRL	DNAJC17
	11	HLA-C*03:02	IVIPFFGLL	IVIPFFSLL	RASGEF1B
	12	HLA-C*03:02	HALAEARHLVY	HAQAEARHLVY	*RCN1**
277	1	HLA-A*11:01	RAIGWQDLARK	RAIGWQDRARK	KDM5D
	2	HLA-A*11:01	RAIGWQDLAR	RAIGWQDRAR	KDM5D
	3	HLA-A*11:01	GTWNRGSSER	GTWNPGSSER	FGA
	4	HLA-A*11:01	RVFERFRRGLK	RVFERFCRGLK	CKMT1A
	5	HLA-A*11:01	MVSWLPGGR	MASWLPGGR	GBGT1
	6	HLA-A*11:01	MVSWLPGGRK	MASWLPGGRK	GBGT1
	7	HLA-A*11:01	TRPSYIRSLQK	TRPSYIRSLRK	PALLD
	8	HLA-A*11:01	DTWPWQVLSK	ETWPWQVLSK	*HEBP1**
	9	HLA-A*11:01	LLGAASSLWAK	LLGAAASLWAK	MEX3D
	10	HLA-A*11:01	ATLALKQFVRR	ATLTLKQFVRR	*LYRM2**
	11	HLA-B*15:02	HPYEHGYPV	QPYEHGYPV	RAD54L2
	12	HLA-B*15:02	HLPAPVHPY	HLPAPVQPY	RAD54L2
	13	HLA-B*15:02	SQWMNDTDCF	NQWMNDTDCF	IPO8
	14	HLA-B*15:02	LALSDLAVL	LALADLAVL	LTB4R
	15	HLA-B*15:02	SDLAVLLTAPF	ADLAVLLTAPF	LTB4R
448	1	HLA-A11:01	QQQPPPPQER	QQPPPPQQER	FBXO11
	2	HLA-A11:01	TVQPPARRMGR	TVQPPARRMGW	MST1
	3	HLA-A11:01	SSIHEAWTDGK	ASIHEAWTDGK	*ACTN4**
	4	HLA-A11:01	VALGPAEKKVK	VALGAAEKKVK	*TNIP1**
	5	HLA-A11:01	KETHLGKVSR	KETHLGKVSL	ZNF550
	6	HLA-A33:03	HKETHLGKVSR	HKETHLGKVSL	ZNF550
	7	HLA-A33:03	TLQDTVLEQDR	TLQDTVLEEDR	ADGRV1
	8	HLA-A33:03	DALKFMHIGKR	DALKFMHMGKR	TAF6
	9	HLA-A33:03	EGIPPDKQR	EGIPPDQQR	**UBC**(4)
	10	HLA-A33:03	FLKGRCKWIR	FLKGSCKWIR	*TMEM97**
	11	HLA-B40:01	KAERHGSRAL	KAERHGSRAR	KDM5C
	12	HLA-B40:01	GKEFSWGAGL	GKEFSWSAGL	ZNF235
	13	HLA-B40:01	GECDPGPEPA	GEGDPGPEPA	TRAPPC12
	14	HLA-B40:01	DELMGLGLHL	DELMGRGLHL	GUCY1A2
	15	HLA-B40:01	KEGIPPDKQRL	KEGIPPDQQRL	**UBC**(4)
	16	HLA-B58:01	RGSPPPPQRL	RGSPPPPQRR	LZTR1
	17	HLA-B58:01	WPIPAYLFSW	GPIPAYLFSW	SLC7A9
	18	HLA-B58:01	QKSSIHEAW	QKASIHEAW	*ACTN4**
	19	HLA-B58:01	YPYLMEAYW	YPYLMEAYG	SLC7A9
	20	HLA-B58:01	RSPILTIITL	RRPILTIITL	*TP53**
	21	HLA-C03:02	RGSPPPPQRLY	RGSPPPPQRRY	LZTR1
	22	HLA-C03:02	QALDSRGSL	QALPSRGSL	FAM163A
	23	HLA-C03:02	FSWGAGLSA	FSWSAGLSA	ZNF235
	24	HLA-C03:02	SAVSTAMKKVF	AAVSTAMKKVF	*MUTYH**
	25	HLA-C07:02	GRLPLLLLL	GLLPLLLLL	MST1
	26	HLA-C07:02	QRRWRPASW	QRRWRPAAW	LAMA3
	27	HLA-C07:02	LRGSPPPPQRL	LRGSPPPPQRR	LZTR1
	28	HLA-C07:02	NRSPILTIITL	NRRPILTIITL	*TP53**
	29	HLA-C07:02	FMHIGKRQKL	FMHMGKRQKL	TAF6
	30	HLA-C07:02	RQYPEIKII	RQYPETKII	*NSD2**
	31	HLA-C07:02	VFPYGLPPL	GFPYGLPPL	*HELZ**(5)

MT: mutated, WT: wildtype
Mtpep: Mutated residues are underlined
* Mutation detected in single cell RNA-seq
(1) LC-MS/MS database VHDLNNDIVKA (AGTPBP1)
(2) LC-MS/MS database TVNQMIARY (SMARCA4)
(3) LC-MS/MS tissue database WFLENKSEVR (METTL2B)
(4) LC-MS/MS database KEGIPPDKQ (UBC)
(5) Only one mutated cell detected in single cell RNA-seq

Table S4. Features of immunogenic peptides in validation experiment

Patient No.	Pep No.	HLA type	Length	MTpep
25	1	HLA-A	10	ILVLGVMVAL
	6#	HLA-A	11	RSPYIPPHSAL
	19*	HLA-C	9	SPILTIITL
	20*	HLA-C	9	LVLGVMVAL
33	2*#	HLA-A	11	SLRRDYYDRGY
	10	HLA-B	11	RPRPTPASTPA
	12	HLA-B	9	WPSLHIEEF
	13	HLA-B	11	CVVAADAYCGW
217	1*	HLA-A	10	GYSKDVLPRL
	2#	HLA-A	11	AVPAPDGQFAF
	3*	HLA-A	11	FFGLLIKDIYF
	5*	HLA-B	11	MGCYDPGRQKW
277	2	HLA-A	10	RAIGWQDLAR
	6	HLA-A	10	MVSWLPGGRK
	7	HLA-A	11	TRPSYIRSLQK
	8#	HLA-A	10	DTWPWQVLSK
	9*	HLA-A	11	LLGAASSLWAK
	10*#	HLA-A	11	ATLALKQFVRR
	13	HLA-B	10	SQWMNDTDCF
	15	HLA-B	11	SDLAVLLTAPF
448	1*	HLA-A	10	QQQPPPPQER
	8	HLA-A	11	DALKFMHIGKR
	17	HLA-B	10	WPIPAYLFSW
	28#	HLA-C	11	NRSPILTIITL
	31#	HLA-C	9	VFPYGLPPL

MT: mutated
* Shared immunogenic peptide
# Mutation detected in single cell RNA-seq

Table S5. Immunogenic potential of peptides with different length and HLA allele

Peptide length	Immunogenicity		Total
	Immunogenic peptide	Non-immunogenic peptide	
9mer	4(13.79%)	25(86.21%)	29
10mer	8(29.63%)	19(70.37%)	27
11mer	13(35.14%)	24(64.86%)	37
Total	25	68	93

Total: Pearson χ2=3.914 *P*=0.141; 9mer VS 11mer: Pearson χ2=3.872 *P*=0.049;
9mer VS 10mer: Pearson χ2=2.083 *P*=0.149; 10mer VS 11mer: Pearson χ2=0.215 *P*=0.643

Peptide HLA-type	Immunogenicity		Total
	Immunogenic peptide	Non-immunogenic peptide	
HLA-A	14(31.82%)	30(68.18%)	44
HLA-B	7(22.58%)	24(77.42%)	31
HLA-C	4(22.22%)	14(77.78%)	18
Total	25	68	93

Total: Pearson χ2=1.036 *P*=0.596; HLA-A VS HLA-B: Pearson χ2=0.77 *P*=0.38;
HLA-A VS HLA-C: Pearson χ2=0.571 *P*=0.45; HLA-B VS HLA-C: Pearson χ2=0.001 *P*=0.977

Supplementary reference

[1] Z. Wei, C. Zhou, Z. Zhang, M. Guan, C. Zhang, Z. Liu, Q. Liu, *iScience* **2019**, *21*, 249, https://doi.org/10.1016/j.isci.2019.10.028.

[2] F. Favero, T. Joshi, A. M. Marquard, N. J. Birkbak, M. Krzystanek, Q. Li, Z. Szallasi, A. C. Eklund, *Ann Oncol* **2015**, *26* (1), 64, https://doi.org/10.1093/annonc/mdu479.

[3] W. Guo, D. Wang, S. Wang, Y. Shan, C. Liu, J. Gu, *Brief Bioinform* **2021**, *22* (3), https://doi.org/10.1093/bib/bbaa127.
